# Recombinant production of amaranthin and other betalain variants with yeast cell factories

**DOI:** 10.1016/j.synbio.2025.05.008

**Published:** 2025-05-21

**Authors:** Christiane Glitz, Jane Dannow Dyekjær, Gian Maria Cristian Solimando, Paulo Marcelo Avila Neto, Daniela Rago, Mahsa Babaei, Irina Borodina

**Affiliations:** The Novo Nordisk Foundation Center for Biosustainability, Technical University of Denmark, Kemitorvet Building 220, DK-2800, Kgs. Lyngby, Denmark

**Keywords:** *Saccharomyces cerevisiae*, *Yarrowia lipolytica*, Glucuronosyltransferase, Natural products, Natural colours, Food science

## Abstract

Betalains are a class of natural pigments found in plants of the Caryophyllales order. Betanin is the dominant betalain on the food colour market, even though over 80 other variants are known. Recombinant production of betanin has recently gained interest as a cost-efficient and sustainable alternative to traditional plant extraction, but the production of other betalain variants remains largely unexplored. We selected three glucuronosyltransferases from *Amaranthus hypochondriacus*, *Chenopodium quinoa* and *Celosia argentea* var. *cristata* and screened the enzymes *in vivo* in betanin-producing *Saccharomyces cerevisiae*. Instead of producing amaranthin, two of the enzymes led to the formation of the betalain bougainvillein-rI (betanidin 5-O-β-sophoroside). When expressed together with a UDP-dehydrogenase that allowed the synthesis of UDP-glucuronic acid, each enzyme converted betanin to amaranthin. Integration of the glucuronosyltransferases in a *Yarrowia lipolytica* betanin producer strain directly resulted in amaranthin production. In fed-batch fermentation, 2.97 g/L ± 29.3 mg/L of amaranthin was produced. Co-expression of the glucuronosyltransferases with a malonyltransferase from *Hylocereus polyrhizus* led to the formation of 6′-O-malonyl-amaranthin in *S. cerevisiae* and *Y. lipolytica*. This study expands the portfolio of natural food colourants that can efficiently be produced through microbial fermentation and contributes to elucidating the biosynthesis pathway of betalains.

## Graphical abstract

**Figure:**
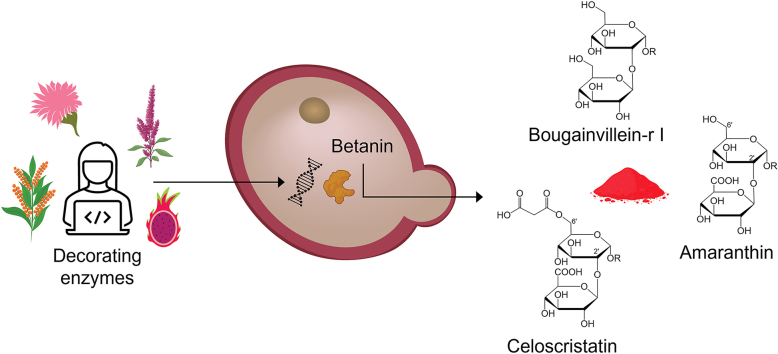

## Abbreviations

AmaSy: Amaranthin synthetase
cDOPA: cyclo-DOPA/Leucodopachrome
GlcAT: Glucuronosyltransferase
MM: Minimal media
SC: Synthetic complete (media)
UGT: UDP-glycosyltransferase

## 1 Introduction

Betalains are a class of natural colourants that exclusively occur in plants of the order *Caryophyllales* and fungi of the genus *Amanita* [1]. These water-soluble, aromatic compounds cover the yellow-to-purple colour spectrum and are divided into two main subgroups: yellow betaxanthins and red betacyanins. A primary source of betalains is beetroot, whose extract is a well-known food colourant marketed as “Beetroot Red” (E162) [2]. This natural dye is commonly used in various products, including confectionery, beverages, meat alternatives, and dairy [3]. Artificial and natural food dyes are widely used to achieve the desired colouration and enhance the overall consumer experience. While artificial dyes have raised concerns about safety and sustainability [[4], [5], [6]], natural colourants are often associated with health-promoting benefits [7]. Betalains, for instance, have been reported to exhibit antioxidant, anti-inflammatory, antimicrobial, anti-diabetic, anti-carcinogenic and anti*-*amyloidogenic properties and to improve cardiovascular health [[8], [9], [10], [11]]. However, the low betanin content in beetroots (<0.5 % wet weight) [12,13] together with a rising demand for natural colours [14], as well as seasonal dependence and the presence of off-flavours and high nitrogen concentrations [15,16] pose significant limitations to the traditional extraction process.

**Fig. 1:**
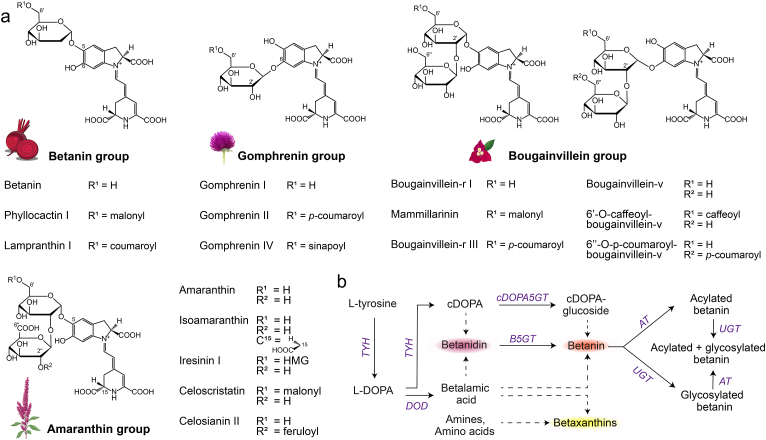
**Groups of betalain variants and their biosynthesis in plants. a** Betalains are categorised into four main groups: betanin, gomphrenin, bougainvillein, and amaranthin group. Examples of structural diversification through acylation, glycosylation and isomerisation. HMG = 3-hydroxy-3-methylglutaryl. **b** Simplified biosynthesis pathway of betaxanthins and betalains in plants. Three enzymes are involved in betanin biosynthesis: a bifunctional tyrosine hydroxylase (TYH), a DOPA-4,5-extradiol dioxygenase (DOD) and a UDP-glycosyltransferase that is either active on cDOPA (cDOPA5GT) or on betanidin (B5GT). Acyltransferases (AT) and UDP-glycosyltransferases (UGT) can modify betanin to produce betalain variants. Dashed arrows represent spontaneous reactions.

Beetroots primarily contain two types of betacyanins: betanin and its isoform isobetanin. In nature, more than 80 betalain variants have been identified [17,18]. This diversity is achieved through modifications of the betanin structure, such as glycosylation, acylation, decarboxylation, or a combination of these modifications [19]. Since beetroots are the primary commercial source of betalains, their diversity on the market is limited to betanin. Betacyanin variants are classified into four groups: betanin-, gomphrenin-, bougainvillein-, and amaranthin-type pigments (Fig. 1a) [20,21]. Unfortunately, most of these modified betalains are present in low concentrations, often in a mix with other betalain variants, and frequently occur in non-commercially used plants such as ornamental flowers, restricting their availability as food dyes [9,18]. This is particularly unfortunate because some modified betalains have demonstrated distinct and partly superior properties regarding stability [22,23], bioactivity [24,25], or hue [19,26]. Stability is a critical factor in the application of food colourants and one of the key drawbacks of natural dyes compared to their synthetic counterparts [27]. While betalains are relatively pH stable, they are susceptible to heat, light, and oxygen, leading to a shorter shelf life of the dyed products [28].

Biotechnological production might be the solution that circumvents some of the disadvantages of plant extraction and provides an opportunity to make more betalain variants commercially available. Heterologous production of other natural colours, such as beta-carotenes and anthocyanins, has already been achieved, and also betanin has been produced in plants, bacteria, and fungi [29,30]. We have previously engineered *S*. *cerevisiae* and *Y*. *lipolytica* to produce betanin by integrating the betanin biosynthetic pathway into these yeast strains (Fig. 1b) [[31], [32], [33]].

Achieving high titres and yields is essential for biotechnological processes to replace the traditional and established extraction methods. The previously mentioned *Yarrowia* strain was engineered to produce up to 1.3 g/L betanin in a fed-batch fermentation [33], and a recent study reported the production of 1.91 g/L betanin in the filamentous fungus *Fusarium venenatum*, to our knowledge the highest betanin titre reported to date [34].

Most studies on heterologous production of betalains have focused on betanin, while production of betalain variants has received little attention. Heterologous production of betalain variants has first been reported in tobacco plants. In one study, aromatically acylated betalain variants such as coumaroyl-betanin and feruloyl-betanin were detected in tobacco plants expressing the betanin pathway genes and a hydroxycinnamoyl-glucosyltransferase from *Mirabilis jalapa*, making use of endogenous acyltransferases from tobacco [35]. Imamura et al. identified the enzymes involved in betalain biosynthesis in *Chenopodium quinoa*, including a GlcAT that glucuronidated betanin to amaranthin. They expressed these enzymes in tobacco plants and BY-2 cell cultures, producing 13.7 μM (9.95 mg/L) amaranthin [24]. Amaranthin is of interest as it serves as a precursor for other derivatives of the amaranthin group, such as celosianin I/II [36], iresinin I [36], mammillarinin [37], and celoscristatin [38] (Fig. 1a). Amaranthin and its derivatives are widely found in species within the Amaranthaceae family, including quinoa (*C*. *quinoa*), various amaranths (e.g. *Amaranthus cruentus*, *A. hypochondriacus*), and cockscombs (e.g. *Celosia argentea*) [9]. We recently reported the microbial production of malonylated betanin, known as phyllocactin, in *S. cerevisiae* and *Y. lipolytica* strains through the expression of an acyltransferase from *H*. *polyrhizus*, reaching 1.95 g/L in fed-batch fermentation [39].

This study aims to expand the portfolio of betalains that can be produced biotechnologically, thereby creating opportunities for variants with potentially improved properties and characteristics that are currently unavailable. Amaranthin, a glucuronidated betalain, serves as the base structure for one of the four main groups of betacyanins, and achieving its production could enable the synthesis of additional variants within the amaranthin group.

## 2 Materials and methods

### 2.1 Assembly and annotation of *Celosia argentea* var. *cristata* transcriptome

We obtained paired-end Illumina HiSeq 2500 transcriptomic RNA-seq raw reads of *C. argentea* var. *cristata* from NCBI (SRR9095475) and generated a fastq file using NCBI's SRAToolKits.2.10.4 fastq-dump tool. We used FastQC to do quality control and check the reads for overrepresented sequences [40]. The reads were trimmed with TrimGalore-0.6.5 using a phred33 quality score [41]. The trimmed, fastq reads were *de novo* assembled using Trinity v2.9.1 [42]. We calculated the assembly statistics with the TrinityStats.pl script in Trinity v2.9.1 and afterwards calculated the expression levels with align_and_estimate_abundance.pl in Trinity v2.9.1 using bowtie as the alignment method and RSEM as the abundance estimation method. Finally, the longest open reading frames (ORFs) were extracted using TransDecoder [43]. Then, we annotated the transcriptome by blasting the transcripts and ORFs into local Uniprot databases. In addition, we extracted the PFAM domains for the ORFs. The results were loaded into an SQLite database using Trinotate v3.2.0 [44,45]. Finally, we merged the expression levels into a final annotation table.

### 2.2 Query for glucuronosyltransferases

To find novel glucuronosyltransferases (GlcATs) that produce glucuronidated betalains, we BLASTed cyanidin-3-O-glucoside 2-O-glucuronosyltransferase from *Bellis perennis* (Daisy, UniProtID: UGAT_BELPE) into the assembled *C. argentea* var. *cristata* transcriptome. The resulting sequence hits were filtered for a length of at least 300 amino acids and presence of the Prosite pattern PS00375. Those hits starting with a start codon were extracted, identical sequences removed and the 10 highest expressed were selected. Three sequences of experimentally validated GlcAT from literature, *Ah*UGT79B30-like4, hereinafter referred to as *Ah*AmaSy1 (from *Amaranthus hypochondriacus*), *Cq*AmaSy1 (from *Chenopodium quinoa*) and *Bv*3GGT-like1 (from *Beta vulgaris*) [24], were BLASTed into the transcriptome of *C. argentea*, and the best hit in a subset of 10 sequences was selected and named *Cc*AmaSy1. *Ah*AmaSy1, *Cq*AmaSy1 and *Cc*AmaSy1 were ordered as gene strings.

### 2.3 Synthetic genes and oligonucleotides

Heterologous genes were synthesised as synthetic gene strings and codon-optimised for *S. cerevisiae* or *Y. lipolytica* by Twist Bioscience (USA). The corresponding amino acid and nucleotide sequences can be found in Table S7. Oligonucleotides were obtained from Integrated DNA Technologies (Table S6).

### 2.4 Media and cultivations

*E. coli* DH5α strains, used for cloning and plasmid propagation, were cultivated in Lysogeny Broth (LB), supplemented with 100 mg/L ampicillin, at 37 °C, 200 rpm. During strain construction, *S. cerevisiae* strains were grown in Yeast Peptone Dextrose media (YPD), supplemented with 200 mg/L Geneticin (G418) for Cas9-plasmid selection, *Y. lipolytica* strains were grown in YPD without G418. After transformation, yeast strains were plated on synthetic complete (SC) agar plates prepared with 76 mg/L of the standard amino acids, 380 mg/L leucine, 18 mg/L adenine, 7 mg/L inositol and 8 mg/L *p*-aminobenzoic acid (*p*ABA). For small-scale cultivations in 24-well plates, mineral media (MM) was prepared with 7.5 g/L (NH_4_)_2_SO_4_, 14.4 g/L KH_2_PO_4_, 0.5 g/L MgSO_4_ 7·H2O, trace metals and vitamins, 20 g/L glucose and adjusted to pH 6.0 with NaOH, as previously described [46]. The yeast strains were cultivated at 30 °C, 250 rpm. For agar plates, 20 g/L agar was added to the respective media.

### 2.5 Plasmid and strain construction

*S. cerevisiae* strains were constructed according to the EasyClone MarkerFree toolkit, *Y. lipolytica* strains were constructed according to the EasyClone YALI toolkit [47,48]. Promoters and heterologous genes were amplified by PCR with USER-compatible overhangs using Phusion U Hot Start DNA Polymerase (Thermo Fisher Scientific, USA) and assembled with PCR-linearised integration vectors in *E. coli* DH5α by USER Cloning [49]. The plasmids were purified from *E. coli* using a NucleoSpin plasmid miniprep kit (Macherey Nagel, Germany), and correct plasmid assembly was verified by Sanger sequencing (Eurofins Genomics, LU). Plasmids were linearised with FastDigest NotI (Thermo Fisher Scientific), resulting in a fragment containing the promoter, gene of interest, terminator and 500–600 bp upstream and downstream homology regions that navigate the integration site in the host genome. The integration fragment and the gRNA were transformed into Cas9-expressing *S. cerevisiae* or *Y. lipolytica* strains with the LiAc method [50]. Transformants were selected on SC plates containing 100 mg/L nourseothricin (*Y.lipolytica*: 250 mg/L nourseothricin) and 200 mg/L G418 (*S. cerevisiae* strains only). Correct integration of the fragment was verified by colony PCR using RedTaq MasterMix (VWR life science).

The *S. cerevisiae* strains used in this study were derived from the haploid strain CEN.PK113-7D (MATa *URA3 HIS3 LEU2 TRP1 MAL2-8c SUC2*). The *Y. lipolytica* strains generated in this study were derived from a W29/CLIB89 (NRRL Y-63746) strain containing a Cas9 expression cassette in the KU70 locus. Glycerol stocks of the created *E. coli* and yeast strains were made by adding glycerol to overnight cultures (25 % (v/v)) and storing them at −70 °C. All biobricks and plasmids created for this work are listed in Tables S4–S5, strains are listed in Table 1 and Table S3.

**Table 1:** Strains used in this study.

Strain	Characteristics	Reference
***Saccharomyces cerevisiae***		

ST7574	CEN.PK113-7D (MATa *URA3 HIS3 LEU2 TRP1 MAL2-8c SUC2)*	[32]
ST9942	ST7574, X-2::tADH1-MjDOD-pTEF1-pPGK1-TYH^W13L^-tCYC1	[32]
ST12160	ST9942, X-3::pTEF1-BvGT2-tADH1	[32]
ST13931	ST12160, XII-5::tADH1-AhAmaSy1-pTEF1	This study
ST13932	ST12160, XII-5::tADH1-CqAmaSy1-pTEF1	This study
ST13946	ST12160, XI-5::tADH1-HpBAHD3-pTEF1	[39]
ST13996	ST12160, XII-5::tADH1-CcAmaSy1-pTEF1	This study
ST14115	ST13931, XI-2::tADH1-AtUGD1-pTEF1	This study
ST14116	ST13932, XI-2::tADH1-AtUGD1-pTEF1	This study
ST14117	ST13996, XI-2::tADH1-AtUGD1-pTEF1	This study
ST14118	ST12160, XI-2::tADH1-AtUGD1-pTEF1	This study
ST14125	ST14115, XI-5::tADH1-HpBAHD3-pTEF1	This study
ST14126	ST14116, XI-5::tADH1-HpBAHD3-pTEF1	This study
ST14127	ST14117, XI-5::tADH1-HpBAHD3-pTEF1	This study

***Yarrowia lipolytica***		

ST6512	W29/CLIB89, MATa ku70Δ::pTEF1-Cas9-tTEF12::pGPD-DsdA-tLIP2	[51]
ST12603	ST6512, IntE1::tPex20-YlARO7^G139S^-pGPD-pTEFin-ARO4^K221L^-tLIP2; IntE3::tPEX20-BvGT2-pTEFin-tPEX20-MjDOD-pGPD-pTEFin-EvTYH-tLIP2; IntE4::tPEX20-BvGT2-pTEFin-tPEX20-MjDOD-pGPD-pTEFin-EvTYH-tLIP2; IntF3::tPEX20-BvGT2-pTEFin-tPEX20-MjDOD-pGPD-pTEFin-EvTYH-tLIP2; Δ4hppd	see ST12376 [33]
ST14100	ST12603, IntC3::pGPD-AhAmaSy1-tLIP2	This study
ST14101	ST12603, IntC3::pGPD-CqAmaSy1-tLIP2	This study
ST14102	ST12603, IntC3::pGPD-CcAmaSy1-tLIP2	This study
ST14103	ST12603, IntD1::pTEFin-HpBAHD3-tLIP2	[39]
ST14760	ST14103, IntC3::pGPD-CcAmaSy1-tLIP2	This study

### 2.6 Small-scale cultivation in 24 well-plates

Screening of the GlcATs in *S. cerevisiae* was performed in 24-deep well plates. Precultures, inoculated from single colonies on agar plates, were grown for 24 h in 2 mL MM (+*p*ABA) in 24-well plates for 24 h. 2 mL MM (without *p*ABA) were inoculated from the precultures in a ratio 1:200 (v/v), corresponding to an OD660 of ca. 0.1, and the strains were cultivated for 48 h at 30 °C and 250 rpm. Afterwards, the cultures’ supernatant and total cell extract (fermentation broth with cells) were analysed. For the supernatant, 1 mL of cultivation broth was centrifuged at 5000 × *g* for 10 min, the supernatant transferred to 1.5 mL reaction tubes, centrifuged again and stored at −20 °C until further use. For the total extract (intracellular + extracellular), 1 mL of cell culture was transferred into a 2 mL microtube (Sarstedt) containing ca. 0.25 mL of 0.5–0.75 mm glass beads. The cells were then disrupted using a Precellys R 24 homogeniser (Bertin Corp.) in five cycles of 5000 rpm for 30 s, with a cooling step on ice between each lysis cycle. After disruption, the tubes were centrifuged for 10 min at 10,000 × *g*, the supernatant transferred to 1.5 mL reaction tubes, centrifuged again and finally stored at −20 °C. The produced titres were not normalised to the OD660 because despite measuring OD at 660 nm instead of 600 nm, there is still overlap with the maximal absorbance of the betacyanins (540 nm), which causes significant deviations in the OD values. The same cultivation protocol was followed to test the enzymes in *Y. lipolytica*, except that the precultures were grown for 48 h.

### 2.7 Fed-batch fermentation

The fed-batch fermentation was carried out in biological replicates using single-use 250 mL bioreactors (AMBR250, Sartorius AG). The *Y. lipolytica* strain ST14102 was streaked from cryostock on YPD-agar plates and incubated for 48 h at 30 °C. 2 mL MM in a pre-culture tube were inoculated with a single colony and incubated with shaking at 250 rpm for 24 h. Afterwards, a baffled shake flask with 50 mL MM was inoculated to OD660 = 0.1 and incubated for another 24 h. The culture was centrifuged (3000 × *g*, 10 min), washed twice with Milli-Q water and concentrated to 10 mL. With this cell suspension, the bioreactors, filled with 100 mL batch media, were inoculated to a starting OD660 of 0.1. The pH was kept at pH 6 with 1 M NaOH, samples were collected automatically every 6 h and immediately frozen (−14 °C). Exponential feeding was initiated once the glucose in the batch media was depleted, where the feed rate F_t_ was adjusted automatically using the equation ${F_{t}=F}_{0}.e^{\mu_{sp}.t}$, with a μ_sp_ set to 0.1. Initial feed rate F_0_ was obtained using equation $F_{0}=C_{X,0\;}.V_{0}.\frac{\mu_{sp}}{Y_{X/S\;}{.\;S}_{f}}$, where C_X,0_ is the concentration of cells after batch phase, V_0_ is the volume in the bioreactor, Y_X/S_ in the yield g_biomass_ per g_substrate_, and S_f_ is the concentration of the glucose in the feed. Afterwards, the glucose concentration in the media and the total betalain concentration (intra- and extracellular) were quantified by HPLC. The fermentation of ST12603 (Run 2) was performed under identical cultivation conditions and followed the same protocol, with the exception of a higher starting OD (0.5 instead of 0.1) and the use of 2 M NaOH for pH control. Medium compositions, operational fermentation parameters and the raw online and offline data can be found as Supporting Information.

### 2.8 Spectrophotometric quantification of betacyanins

As reported before, betanin and isobetanin were quantified using a commercially available beetroot extract (TCI, Product Number: B0397) [31]. Since no commercial standards for bougainvillein-r I and amaranthin were available, standard solutions were prepared in-house. Dried *Bougainvillea glabra (*pink*)* and *Amaranthus cruentus* (dark red) flowers were ground with 10 mM ascorbic acid with mortar and pestle until the liquid was deeply red-coloured. Solid plant residues were removed by centrifugation (4 °C, 11,000 × *g*, 5 min), followed by filtration through a 0.45 μm syringe filter, then a 0.2 μm syringe filter. The extracts were stored at −20 °C until analysis by HPLC and LC-MS. For quantification, bougainvillein-r I and amaranthin were purified from the *B*. *glabra* and *A. cruentus* extracts, respectively, using semi-preparative HPLC. Absorbance at 535 nm was measured in a spectrophotometer. To determine the concentration of the pigments, Beer-Lambert equation was employed: c _(betalain mg/L)_ = (*A* x MW x *DF* x 1000) x (ɛ x *l*)^−1^, whereby c = concentration of the betalain in mg/L, *A* is the measured absorbance at 535 nm, MW is the molecular weight of the betalain, *DF* is the dilution factor, ɛ is the molar extinction coefficient (6.5 × 10^4^ M^−1^ cm^−1^) and *l* is the path length of the cuvette (1 cm). Calibration curves were made assuming the same molar extinction coefficient for all betalain variants (Table S1).

### 2.9 Analytical methods

To quantify the betalains produced by the yeast cultures, the samples were analysed via high-performance liquid chromatography (HPLC) using a Dionex Ultimate 3000 HPLC system (Thermo Fisher Scientific, US). The samples were run on a Zorbax Eclipse Plus C18 reverse-phased column (particle size 3.5 μm, pore size 95 Å, 4.6 × 100 mm). The column oven temperature was set to 30 °C and the flow rate to 1 mL/min, with 10 μL of sample injection. The mobile phase consisted of Solvent A (0.1 % formic acid), and Solvent B (100 % acetonitrile). The solvent composition was initially set to 98.0 % of Solvent A and 2.0 % of Solvent B and was kept steady for 2 min. Afterwards, a linear gradient was run until 90.0 % of Solvent A was reached at 5.0 min. Then, the slope of the gradient was decreased until reaching 85.0 % of Solvent A at 8.0 min. At 8.2 min, the column was flushed by setting Solvent A to 2.0 % and Solvent B to 98.0 %. At 9.5 min, the initial conditions (A = 98.0 %, B = 2.0 %) were reset and remained unchanged until the end of the run (11.5 min). The UV–Vis-detector was set to capture data at 390 nm, 480 nm and 540 nm.

Semi-preparative HPLC was performed to obtain pure fractions of bougainvillein-r I and amaranthin for quantification. Per run, 100 μL of sample were injected into a Zorbax Eclipse Plus C18 reverse-phased column (particle size 3.5 μm, pore size 95 Å, 4.6 × 100 mm) that was kept at 30 °C and run with a flow rate of 0.8 mL/min. Solvent A was composed of water + 0.1 % formic acid, Solvent B of 100 % acetonitrile. The solvent composition was set to 98.0 % of Solvent A and 2.0 % of Solvent B, and kept steady for 2 min. Hereafter, the solvent composition was adjusted following a linear gradient until solvent A reached 90.0 % at 12 min. Then, the solvent composition was adjusted following a second linear gradient until reaching 80.0 % of Solvent A at 17 min. The column was then flushed with 98 % Solvent B at 18 min. These conditions were kept steady until 18.5 min and were then returned to the initial conditions at 19 min, at which point the solvent composition remained unchanged until the end of the run at 20.5 min. All betalains were detected with a UV-VIS detector at a wavelength of 540 nm. To obtain the desired compound fraction, the respective time span in which that compound elutes was selected. The acetonitrile was evaporated, and the pooled fractions of multiple runs up-concentrated with a vacuum evaporator to achieve higher concentrations. Afterwards, the fraction was analysed by LC-MS for validation and then used as HPLC standards to make a calibration curve.

For glucose quantification, the HPLC system was equipped with an Aminex organic acid column kept at 50 °C. Isocratic elution was run with 5 mM H_2_SO_4_ at a flow rate of 0.6 mL/min for 30 min. Glucose was detected with a RI detector, 20 μL were injected.

Untargeted LC–UV–tandem mass spectrometry (MS^2^) analysis was performed to identify the betalain variants in plant extracts and yeast cultivations, using a Vanquish Flex UHPLC binary system (Thermo Fisher Scientific, USA) coupled to a DAD-(ESI)Fusion Orbitrap Mass Spectrometer (Thermo Fisher Scientific, USA). The chromatographic separation was achieved using a Waters ACQUITY BEH C18 column (10 cm × 2.1 mm, 1.7 μm) equipped with an ACQUITY BEH C18 guard column at 30 °C and a flow rate of 0.35 mL/min. The mobile phase was composed of Solvent A (Milli-Q water + 0.1 % formic acid) and Solvent B (acetonitrile + 0.1 % formic acid) and run with a gradient as follows: Solvent B was at 0.2 % for 3 min, followed by a linear increase up to 25 % within 20 min and kept steady for 1 min. After that, the concentration of Solvent B increased to 100 % in 4 min and stayed at 100 % for 2 min before returning to initial conditions. Re-equilibration time was 2 min, 1μL sample was injected. The DAD settings were the following: data collection rate: 10 Hz, wavelength range: 190–600 nm, bandwidth: 2 nm. MS acquisition was set to positive-heated electrospray ionisation (+HESI) mode with a voltage of 3500 V, acquiring in full MS^2^ spectra (data-dependent acquisition-driven MS^2^) with a mass range of 70–1000 Da. DAD acquisition settings were the following: automatic gain control (AGC) target value set at 4e5 for the full MS and 5e4 for the MS^2^ spectral acquisition, MS1 resolution was set to 120,000 and 30,000 for MS^2^ events. Precursor ions were fragmented by stepped high-energy collision dissociation (HCD) using collision energies of 20, 40, and 60. A summary of the detected betacyanin pigments can be found in Table S2.

The quantification of UDP-glucose and UDP-glucuronic acid was performed by LC-MS with the same instrumentation as described above and utilising commercial standards. The compounds separation was achieved using a Waters Acquity UPLC BEH Amide (10 cm × 2.1 mm, 1.7 μm) column equipped with an Acquity UPLC BEH amide guard column kept at 40 °C. The mobile phase composition consisted of Solvent A (MilliQ water + 10 mM ammonium acetate adjusted with ammonium hydroxide to reach pH 9.2) and Solven B (acetonitrile + 0.1 % formic acid). The chromatographic conditions used were as the ones previously described [52]. The MS^2^ acquisition was done in negative-HESI applying a voltage of 2500 V and using the same settings as described above. Thermo Xcalibur Quant Browser Software (Thermo Fisher Scientific, USA) was used for the peak integration of UDP glucose (*m*/*z* 565.0473) and UDP glucuronic acid (*m*/*z* 579.0264) allowing 5 ppm as *m*/*z* tolerance. Calibration curve of each compound in the linear range of 10–50 μM were applied for compound quantification.

## 3 Results and discussion

### 3.1 Screening of glucuronosyltransferases in betanin-producing *S. cerevisiae*

To produce amaranthin from betanin, a glucuronosyltransferase (GlcAT) is required to attach glucuronic acid to the C2′ position of betanin or cDOPA-glucoside. Imamura et al. identified a flavonoid glucosyltransferase from quinoa (*Cq*AmaSy1) capable of synthesising amaranthin when co-expressed with betanin biosynthesis genes in *N. benthamiana* and tobacco BY-2 cells. By phylogenetic analysis, they found two additional GlcATs: *Bv*3GGT-like1 from *Beta vulgaris* and *Ah*UGT79B30-like4 (hereinafter referred to as *Ah*AmaSy1) from *Amaranthus hypochondriacus*, both of which also led to amaranthin accumulation when expressed in tobacco leaves. These enzymes have not been previously expressed in microbial systems. Cockscomb (*Celosia argentea*) is known for producing high levels of amaranthin and its acylated derivatives celosianin I and II [38,53], making the flower a promising source for GlcATs with high activity. We assembled and annotated the publicly available *Celosia argentea* var. *cristata* transcriptome and BLASTed a GlcAT (cyanidin-3-O-glucoside 2-O-GlcAT) from *Bellis perennis*, involved in anthocyanin glucuronidation, against it. Additionally, the by Imamura et al. experimentally validated GlcATs *Ah*AmaSy1, *Cq*AmaSy1, and *Bv*3GGT-like1 were BLASTed into the *C. argentea* transcriptome and the best hit, which was one of the ten highest expressed GlcATs, was named *Cc*AmaSy1. Of those AmaSy candidate genes, we chose to test *Ah*AmaSy1, *Cq*AmaSy1 and *Cc*AmaSy1 for amaranthin formation in *S. cerevisiae*. *Bv*3GGT-like1 was excluded because Imamura et al. demonstrated that, when expressed in *N. benthamiana* leaves, it was less efficient at converting betanin to amaranthin than *Ah*AmaSy1 and *Cq*AmaSy1.

**Fig. 2:**
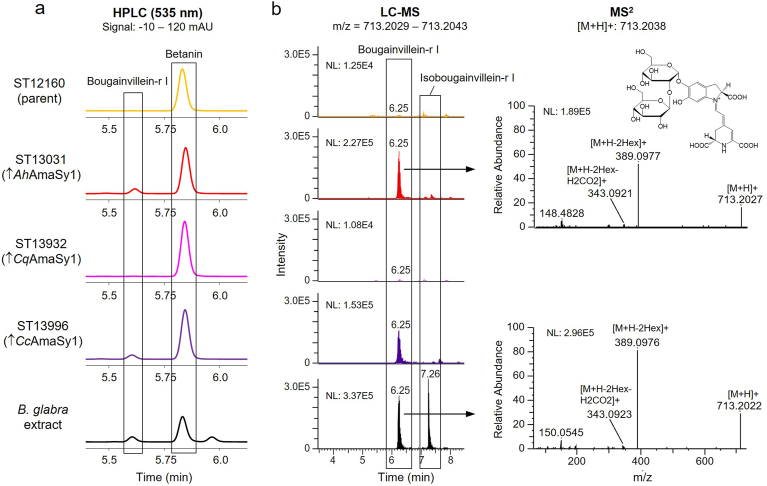
**Screening of glucuronosyltransferases in betanin-producing *S. cerevisiae*. a** HPLC-UV chromatogram, comparing the total fraction of the parent strain ST12160 with the strains expressing *Ah*AmaSy1, *Cq*AmaSy1 or *Cc*AmaSy1 and the plant extract from *B. glabra*. A compound with an RT of 5.4 min, also present in the plant, was detected in the strains expressing *Ah*AmaSy1 and *Cc*AmaSy1. The upward arrow (↑) indicates overexpression of the gene listed after the symbol. **b** Extracted-ion chromatogram (XIC) and the corresponding MS^2^ spectrum from the LC-MS analysis revealed that this new compound with *m*/*z* = 713.2039 is bougainvillein-r I. The product ion at *m*/*z* = 389.09, characteristic for all betacyanins, corresponds to betanidin, the product ion at *m*/*z* = 343.09 corresponds to monodecarboxylated betanidin.

We have previously constructed a betanin-producing *S. cerevisiae* strain (ST12160), expressing the betanin biosynthesis genes *Bv*TYH^W13L^, *Mj*DOD and *Bv*GT2 [31]. This strain, which produced 6.8 mg/L betanin in small-scale cultivations, was used as parent strain for screening of the GlcATs. Integration of the AmaSy genes into ST12160 created strains ST13931, ST13932 and ST13996. The yeast strains were cultivated in 2 mL MM for 48 h. Afterwards, the supernatant and total extract (intra- and extracellular fractions) were analysed by HPLC. The HPLC chromatogram showed a small peak with a retention time (RT) of 5.4 min and *λ*_max_ of 537 nm in those strains expressing *Ah*AmaSy1 and *Cc*AmaSy1, which was neither present in the parent strain nor in the strain expressing *Cq*AmaSy1 (Fig. 2a). A slightly lower retention time than betanin matched the reported time for amaranthin [38,54], and we thus assumed that the strains produced the glucuronidated betanin. However, LC-MS analysis of the cultivation broth with a plant extract from *Amaranthus cruentus* showed that the produced compound was not amaranthin (Fig. S1). Instead, the detected compound had a *m*/*z* of 713.2039 ([M+H]^+^). Comparison of the LC-MS and MS^2^ profile with a plant extract from *Bougainvillea glabra* (Fig. 2b) confirmed our suspicion that the produced compound was betanidin 5-O-β-sophoroside, also known as bougainvillein-r I, one of the betalain variants found in members of the genus *Bougainvillea* [55].

Almost all betalains occur together with their C15 stereoisomers. The isoforms (e.g. isobetanin) usually have a slightly higher RT than the “normal” form, as is reported for bougainvillein-r I and isobougainvillein-r I [56]. Therefore, we concluded that our strains produced primarily bougainvillein-r I while the plant also produced isobougainvillein-r I.

### 3.2 Expression of *At*UGD1 enables amaranthin production in *S. cerevisiae*

**Fig. 3:**
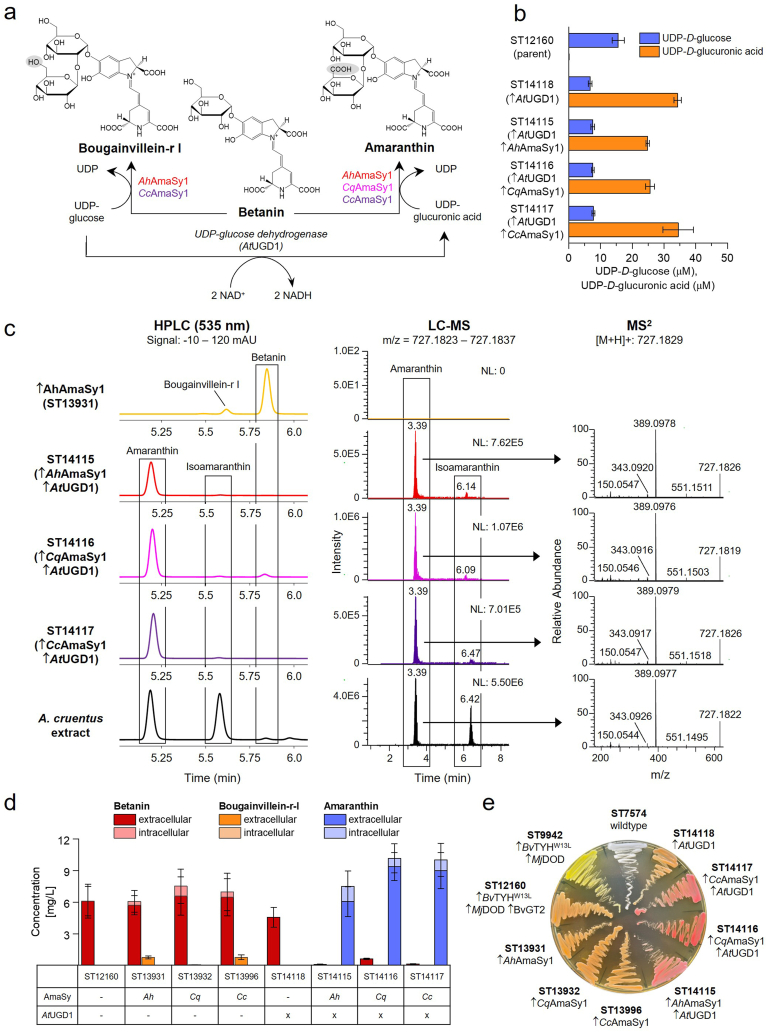
**UDP-glucuronic acid synthesis in *S. cerevisiae* enables the production of amaranthin by glucuronosyltransferases**. **a** Depending on the available sugar donor, betanin is glycosylated to bougainvillein-r I or amaranthin by the glucuronosyltransferases. *At*UGD1 converts UDP-glucose to UDP-glucuronic acid. **b** UDP-glucose and UDP-glucuronic acid concentrations in betalain producing *S. cerevisiae* without (ST12160) and with expression of a heterologous UDP-glucose dehydrogenase (*At*UGD1). **c** HPLC analysis detects a new compound in all three strains expressing *At*UGD1 in combination with a glucuronosyltransferase that is also present in the plant extract from *A. cruentus*. LC-MS analysis confirms that this compound is amaranthin. **d** Betalain production in strains with or without *At*UGD1, quantified by HPLC. Concentrations of C15 stereoisomers were combined. Mean values of biological triplicates (±SD) are shown. “X” indicates the integration of *At*UGD1. **e** Betalain-producing *S. cerevisiae* strains on agar plate. *At*UGD1: UDP-glucose dehydrogenase (*A. thaliana*), *Ah*AmaSy1: Glucuronosyltransferase (*Amaranthus hypochondriacus*), *Cq*AmaSy1: Glucuronosyltransferase (*Chenopodium quinoa*), *Cc*AmaSy1: Glucuronosyltransferase (*Celosia argentea* var. *cristata*), *Mj*DOD: 4,5-extradiol dioxyge*nase* (*Mirabilis jalapa*), *Bv*TYH: tyrosine hydroxylase (*Beta vulgaris*), *Bv*GT2: UDP-glycosyltransferase (*Beta vulgaris*). The upward arrow (↑) indicates overexpression of the gene listed after the symbol.

It was intriguing that the enzymes facilitating amaranthin production in tobacco either did not produce any betacyanin besides betanin (*Cq*AmaSy1) or produced only small amounts of bougainvillein-r I (*Ah*AmaSy1, *Cc*AmaSy1) when expressed in *S. cerevisiae*. Bougainvillein-r I and amaranthin are structurally similar betacyanins, differing only by the sugar attached to the C2′ position of betanin: glucuronic acid in amaranthin and glucose in bougainvillein-r I. *Ah*AmaSy1, *Cq*AmaSy1 and *Cc*AmaSy1 belong to the superfamily of UDP-glycosyltransferase (UGT), which requires an activated nucleotide diphosphate sugar, e.g. UDP-glucose or UDP-glucuronic acid, to transfer a sugar moiety to the acceptor molecule such as betanin [57]. *S. cerevisiae* is known to have a substantial cytoplasmic pool of UDP-glucose (∼2 μmol/g dry cell weight) but we were unsure if the yeast also produced UDP-glucuronic acid. After some literature research, we discovered that it lacks the endogenous capacity to synthesise UDP-glucuronic acid, explaining why expression of the GlcATs did not lead to amaranthin production (Fig. 3a) [58,59].

Oka and Jigami engineered *S. cerevisiae* to synthesise UDP-glucuronic acid *de novo* by introducing the UDP-glucose dehydrogenase *At*UGD1 from *Arabidopsis thaliana*, which efficiently catalysed the conversion of UDP-glucose into UDP-glucuronic acid [60]. *At*UGD1 was integrated into the parent strain ST12160, resulting in ST14118, and the intracellular UDP-glucose and UDP-glucuronic acid concentrations were measured by LC-MS (Fig. 3b). While the parent strain produced 15.6 μM UDP-glucose and no detectable UDP-glucuronic acid, integration of *At*UGD1 increased the UDP-glucuronic acid pool to 34 μM in ST14118. At the same time, UDP-glucose concentration decreased but was not depleted. Building on these promising results, three strains were created that expressed *At*UGD1 together with one GlcAT: ST14115, expressing *Ah*AmaSy1 and *At*UGD1, ST14116, expressing *Cq*AmaSy1 and *At*UGD1 and ST14117, expressing *Cc*AmaSy1 and *At*UGD1.

Already on agar plate, the strains with integrated *At*UGD1 and AmaSy were distinct from the other strains by their pink-red colour (Fig. 3e). Cultivation of the strains in 24-well plates and subsequent analysis of extra- and intracellular fractions by HPLC confirmed the production of large amounts of a compound with an RT of 5.17 min and *λ*_max_ of 537 nm by all three GlcATs that was also present in the extract from *A. cruentus* (Fig. 3c). The samples were subsequently analysed by LC-MS and an *m*/*z* of 727.1829, and the MS^2^ fragmentation pattern confirmed that the produced compound was indeed amaranthin (Fig. 3c). The strains also produced small amounts of isoamaranthin, the C15 stereoisomer of amaranthin, which had a similar retention time as bougainvillein-r I in the HPLC (5.58 min) but was distinguishable by LC-MS. Evidently, without UDP-glucuronic acid, *Ah*AmaSy1 and *Cc*AmaSy1 can catalyse the glycosylation of betanin to bougainvillein-r I, while *Cc*AmaSy1 does not show this promiscuity. If UDP-glucuronic acid is available, all three GlcATs are selective for amaranthin formation.

We quantified the betalains produced by the yeast strains with and without integrated *At*UGD1 based on the HPLC results (Fig. 3d). Concentrations of bougainvillein-r I and amaranthin were determined by applying the Beer-Lambert equation to pure bougainvillein-r I and amaranthin, purified by semi-preparative HPLC from flowers of *B. glabra* and *A. cruentus,* respectively. The highest amaranthin titre of 10.2 ± 2.7 mg/L (9.4 mg/L intracellular, 0.8 mg/L intracellular) was reached with *Cq*AmaSy1, but also *Ah*AmaSy1 and *Cc*AmaSy1 produced >7.5 mg/L amaranthin. Only traces of betanin were detected in all three strains, indicating a high efficiency of the enzymes to glucuronidate betanin. Compared to those, the strains without UGD produced minimal amounts of bougainvillein-r I, the highest being 0.76 mg/L with *Cc*AmaSy1. This suggests that the enzymes exhibit very low catalytic efficiency toward UDP-glucose, leading to minimal production of bougainvillein-r I. In contrast, *Cq*AmaSy1 exhibited strict specificity, exclusively utilising UDP-glucuronic acid as its sugar donor.

As reported, most of the betanin was found in the extracellular fraction [31]. The same trend was observed for amaranthin (80–92 % secreted) and bougainvillein-r I (100 % secreted). So far, we have not identified the transporter(s) involved in the export of betanin and other betalain variants. However, the data suggested that their transport efficiency remained uncompromised despite amaranthin and bougainvillein-r I being larger than betanin.

It has been debated for some time whether the acceptor molecule for the glucuronidation is betanin or cDOPA 5-O-glucoside. Sasaki et al. reported in 2005 that in feather cockscomb, glucuronic acid is conjugated to cDOPA-glucoside but not to betanin [61]. Previously, feeding experiments of betacyanin precursors to *Celosia plumosa* seedlings showed that cDOPA-glucoside is a better precursor for amaranthin synthesis than betanidin or betanin [62]. Imamura et al. analysed the substrate specificity of *Cq*AmaSy1 in an *N. benthamiana* transient expression system by expressing *Cq*AmaSy1 in combination with a betanidin-5-GT that can only produce betanin but not cDOPA-glucoside [24]. The presence of amaranthin in the leaves indicated that *Cq*AmaSy1 synthesises amaranthin using betanin as substrate. This finding aligns with what has been observed for anthocyanins, where the glucuronic acid is transferred to the anthocyanidin-glucoside [63]. The UGT we use for betanin biosynthesis in yeast, *Bv*GT2, clusters with 5-O betanidin GT and has been shown to form betanin via glycosylation of betanidin [31,64]. Whether *Bv*GT2 can also glycosylate cDOPA could not fully be determined. The high amaranthin concentrations reached by the strains in this experiment suggested that the tested GlcATs can glucuronidate betanin and not or at least not exclusively cDOPA-glucoside.

Based on the production of amaranthin in betanin-producing *S. cerevisiae*, we assumed that *Cc*AmaSy1 is also involved in betalain biosynthesis in *Celosia argentea* var. *cristata* and either alone or with other GlcATs responsible for producing amaranthin and its derivatives in the *Amaranthaceae*.

### 3.3 Expression of glucuronosyltransferases in *Yarrowia lipolytica* leads to efficient amaranthin production

**Fig. 4:**
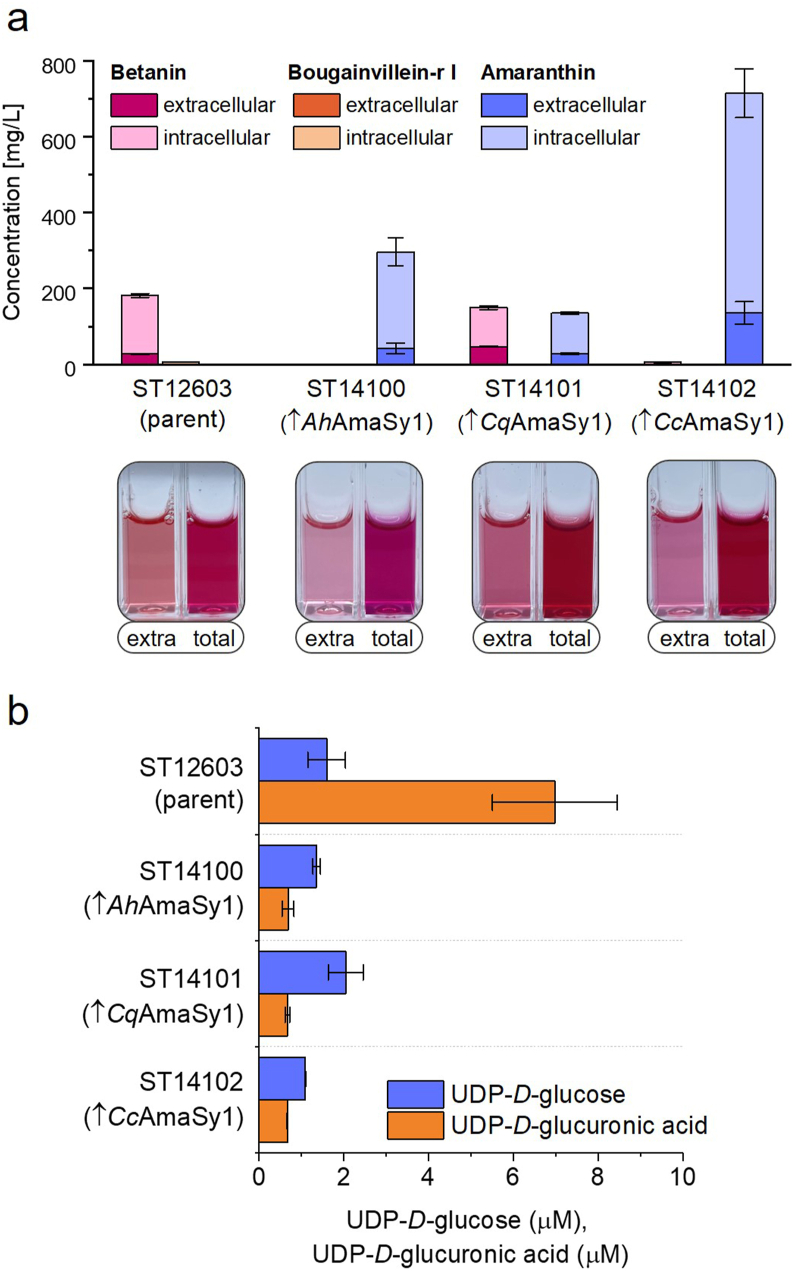
**Expression of glucuronosyltransferases in betanin-producing *Y. lipolytica*.** ST12603 and the GlcAT-expressing strains were cultivated in 24-well plates for 48 h. **a** Intra- and extracellular betalain concentrations, determined by HPLC. The corresponding 10x diluted samples are shown below the graph. Concentrations of the C15 stereoisomers were combined. Mean values of biological triplicates (±SD) are shown. **b** UDP-glucose and UDP-glucuronic acid concentrations in the yeast strains were measured by LC-MS. The upward arrow (↑) indicates overexpression of the gene listed after the symbol.

We recently developed a *Yarrowia* strain (ST12603) that produced high betanin titres. This strain was engineered towards betanin production by integrating three copies of the biosynthetic pathway (*Mj*DOD-*Ev*TYH-*Bv*GT2), introducing feedback-inhibition-resistant Aro4 (*Yl*ARO4K221L) and Aro7 (*Yl*ARO7G139S), and deleting the 4-hydroxyphenylpyruvate acid dioxygenase (Δ4HPPD) to prevent by-product formation. After confirming that all three GlcATs were active in *S. cerevisiae*, we were curious how these enzymes would perform in the high-producing *Yarrowia* strain and whether the strain would produce amaranthin or bougainvillein-r I. One copy of each GlcAT, codon-optimised for *Yarrowia*, was integrated into the platform strain ST12603, resulting in strains ST14100 (expressing *Ah*AmaSy1), ST14101 (expressing *Cq*AmaSy1) and ST14102 (expressing *Cc*AmaSy1). The strains were tested in a small-scale cultivation in 2 mL MM. HPLC (Fig. 4a) and LC-MS analysis (Fig. S2) of the strains and the plant extract from *A. cruentus* confirmed the production of amaranthin and isoamaranthin by all three strains. Interestingly, small amounts of bougainvillein-r I were detected in the parent strain ST12603 but in none of the strains expressing a GlcAT. As expected, the engineered parent strain ST12603 produced significantly more betanin than the *S. cerevisiae* strain ST12160, which expressed only one copy of the biosynthesis pathway. Each of the GlcATs led to the conversion of betanin into amaranthin. One copy of *Ah*AmaSy1 and *Cc*AmaSy1 was sufficient to glucuronidate all betanin so that no betanin was left in the respective strains. In total, ST14100 produced 296 ± 52 mg/L (0.41 ± 0.07 mM) amaranthin while ST14102 produced 715 ± 94 mg/L (0.98 ± 0.13 mM) amaranthin (Fig. 4a). The GlcAT from *C. quinoa,* which performed best in *S. cerevisiae*, could not convert all the betanin to amaranthin, resulting in a lower amaranthin titre of 135 ± 5 mg/L (0.19 ± 0.007 mM). The different titres were also reflected in the colour of the samples. ST14100 and ST14102 showed a more intense and purple colour than the parent strain and ST14101. As seen for betanin, most amaranthin was detected inside the cells, and only 14–21 % of it was secreted into the media. We did not investigate the underlying reasons for the substantial differences in titres among the three strains and can thus only speculate. Possible factors include differences in expression levels, protein folding or subcellular localisation, as well as aspects of enzyme kinetics such as substrate specificity or turnover rates.

From the observed production of amaranthin, we concluded that *Y. lipolytica*, in contrast to *S. cerevisiae*, is endogenously capable of synthesising UDP-glucuronic acid. To verify, the UDP-glucose and UDP-d-glucuronic acid concentrations in the *Yarrowia* strains were determined (Fig. 4b). As expected, both UDP-sugars were present in the yeast. The betanin-producing strain ST12603 contained 7 μM UDP-glucuronic acid but only 1.6 μM UDP-glucose. The amount of UDP-sugars produced by the wild-type strain W29, which lacks any betanin biosynthesis pathway genes, was not determined. In the amaranthin-producing strains, the UDP-glucuronic acid content decreased from 7 μM to around 0.68 μM, while the UDP-glucose content was comparable to the parent strain. Even though the UDP-glucuronic acid pool was not entirely depleted, the low concentrations suggest that its supply is limiting for amaranthin production.

This experiment showed that all three GlcATs were active in *Yarrowia* and demonstrated their efficiency in converting betanin to amaranthin. Given that amaranthin formation requires an additional molecule of UDP-glucuronic acid compared to betanin, it was remarkable that amaranthin titres exceeded betanin titres by 1.6-fold with *Ah*AmaSy1 and 3.9-fold with *Cc*AmaSy1.

### 3.4 Gram-scale production of amaranthin in fed-batch fermentation

**Fig. 5:**
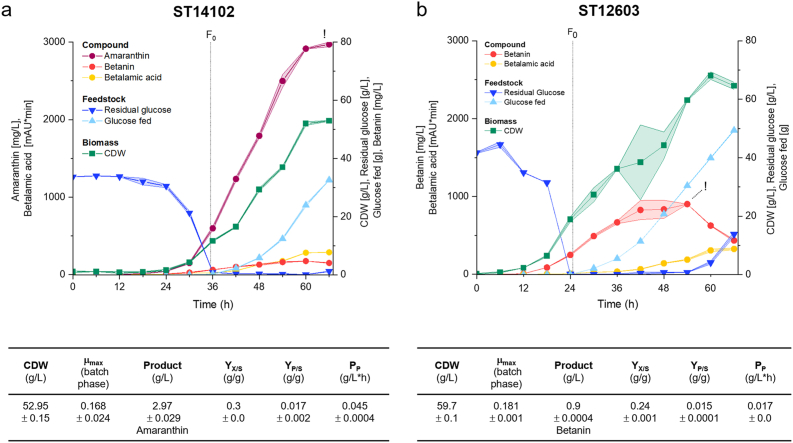
**Fed-batch fermentation in bioreactor**. *Y. lipolytica* strain ST14102 and its parent strain ST12603 were cultivated in 250 mL AMBR bioreactors in duplicates, run for 66 h. **a** Amaranthin-producing strain ST14102. pH control: 1 M NaOH, inoculation: OD 0.1 (Run 1). **b** Betanin-producing ST12603. pH control: 2 M NaOH, inoculation: OD 0.5 (Run 2). Solid lines indicate the average from both bioreactors, shaded areas represent the corresponding standard deviations. F_0_ = feed initiation. The tables list the relevant process data of each run at the timepoint of the highest betacyanin titre (!).

After the promising results in small-scale, the *Cc*AmaSy1 expressing strain ST14102 and its betanin-producing parent strain ST12603 were tested in a fed-batch fermentation (Run 1). The fermentations were carried out in biological duplicates in 250 mL AMBR bioreactors in MM at pH 6 with glucose as carbon source. Only the total betacyanin production was determined. Unfortunately, both replicates of ST12603 were aborted after 35 h and 40 h of cultivation due to excessive foaming and the delayed automatic addition of antifoam by the AMBR250 system, making them unsuitable references. Therefore, data from an additional fed-batch fermentation of ST12603, conducted under similar process conditions and hereafter referred to as Run 2, was used for comparison. The only differences between the setups were the base concentration (1 M NaOH in Run 1 vs. 2 M NaOH in Run 2) and the inoculation OD (OD 0.1 in Run 1, OD 0.5 in Run 2). Both fermentations employed exponential feeding to maintain a specific growth rate of 0.1 h^−1^ during the feed phase. Betacyanin titres, feedstock development and biomass formation for both strains are shown in Fig. 5.

Compared to the cultivation of ST14102, ST12603 exhibited a considerably shorter lag phase, allowing feeding to begin after 24 h, 12 h earlier than with ST14102. Based on other cultivations, we consider it more likely that this difference is due to the specific ST14102 preculture and the lower starting OD rather than a general trend between the strains. The fermentation of the amaranthin-producing strain ran for 66 h, reaching a final titre of 2.97 ± 0.029 g/L amaranthin, with both production and biomass formation slowing down after 60 h. In the betanin-producing strain, production peaked after 54 h with 900 mg/L betanin. The subsequent decrease in betanin titre is likely due to a combination of degradation and dilution effect resulting from feeding and base addition. This effect is also relevant for amaranthin, where the dilution impact is expected to be even more pronounced due to the lower base concentration and high base volume needed to neutralise the formation of acidic product. Relevant process parameters at the time of highest betacyanin titres are compared in the tables in Fig. 5. Under the given conditions, amaranthin production outperformed that of the betanin-producing strain, not only by achieving a threefold higher titre (2.97 g/L vs. 0.9 g/L betanin), but also in terms of yield and productivity.

The raw data for the fermentations can be found in SI File 2, the online process data in Fig. S3. The HPLC chromatograms for ST14102 at 540 nm, 480 nm and 410 nm are depicted in Fig. S4.

The physicochemical properties of amaranthin have received little attention in the past, but the existing reports suggest that pure amaranthin is either as stable or less stable than betanin during thermal treatment, at high oxygen concentrations and across various pH levels [[22], [65]]. It can be hypothesised that the high production shown here is due to enhanced stability during cultivation, such as increased resistance to enzymatic degradation or the facilitation of the compound's storage in the vacuole. Additionally, it is known that plants employ glycosylation as a mechanism for detoxification and the glucuronidation might have the same effect in the yeast strains as well, becoming relevant when high betalain concentrations are being produced [66,67].

To further increase the amaranthin titres, both optimisation of the fermentation process and strain engineering strategies should be pursued. For the latter, integration of a second GlcAT copy should be considered. As *Y. lipolytica* can convert UDP-glucose into UDP-glucuronic acid, it must possess an enzyme that catalyses this reaction. In the genome of the here-used *Y. lipolytica* wildtype strain W29 or CLIB89, the hypothetical protein YALI1_DO2781g shows high sequence similarity to the UDP-glucose dehydrogenase UGD1p from the yeast *Cryptococcus neoformans* (Q96VU5). In *Y. lipolytica* CLIB122, the gene is annotated as UDP-alpha-d-glucose:NAD+6-oxidoreductase (YALI0D02321g). The low UDP-glucuronic acid concentrations in the amaranthin-producing strains suggested that the UDP-sugar was depleted at the end of the cultivations. Combined with strategies to increase the UDP-glucose titre, overexpression of this putative UGD1 might thus be a promising engineering target to boost amaranthin production further. Since no betanin was left after cultivation, betanin production seemed to be another bottleneck of the process. Further addition of biosynthetic pathway genes has not been shown to increase titres [33]. However, optimising the balance of enzymes within the pathway could have a beneficial effect. Other promising targets for strain engineering include enhancing precursor and cofactor supply, as well as disrupting degrading enzymes as demonstrated by the deletion of beta-glucosidases by Thomsen et al. [33]. To evaluate the potential of amaranthin as an alternative to betanin as a food colourant, further characterisation of the pigment and its stability is required.

A life cycle analysis and techno-economic assessment (TEA) previously demonstrated that the biotechnological production of betanin is not only more sustainable than traditional extraction from beet roots, but that even a titre of 1.1 g/L enables economically feasible production [33]. The TEA further showed that a threefold increase in titre (compared to 1.1 g/L) could reduce production costs by 34.5 %. Given the nearly 3 g/L amaranthin obtained here with a superior yield (Y_P/S_), the production of amaranthin can be assumed to be economically feasible for industrial applications under current market conditions.

### 3.5 Co-expression of a glucuronosyltransferase and BAHD acyltransferase leads to formation of malonylated amaranthin in betanin-producing yeast

**Fig. 6:**
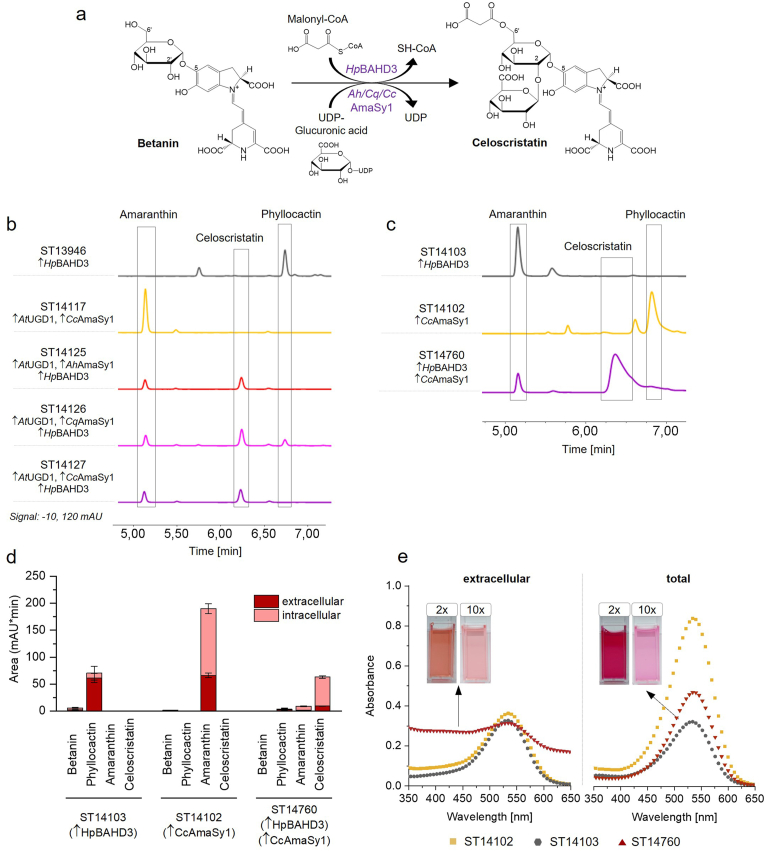
**Celoscristatin production in *S. cerevisiae* and *Y. lipolytica*. a** To synthesise malonylated amaranthin from betanin, a glucuronosyltransferase (*Ah*/*Cq*/*Cc*AmaSy1) and a malonyltransferase (e.g. *Hp*BAHD3) are required. **b** HPLC chromatogram (540 nm) of the extracellular fraction of amaranthin-producing *S. cerevisiae* strains, additionally expressing the acyltransferase *Hp*BAHD3. A new compound was detected in all three strains, absent in the reference strains. Y-axis: 10 – 120 mAU. The upward arrow (↑) indicates overexpression of the gene listed after the symbol. **c** Expression of *Hp*BAHD3 and *Cc*AmaSy1 in betanin-producing *Y. lipolytica* led to the formation of the same compound. From LC-MS analysis, this new compound was identified as celoscristatin (6′-O-malonyl-amaranthin) (Fig. S5a). **d** Phyllocactin- (ST14103), amaranthin- (ST14102) and celoscristatin- (ST14760) producing *Y. lipolytica* strains were cultivated in MM and the extracellular and intracellular production analysed by HPLC. Peak areas (mAU∗min) were compared. Mean values of biological triplicates (±SD) are shown. **e** UV–vis spectrum of 10x diluted extracellular and total samples. The corresponding 2x and 10x diluted samples of ST14760 are shown.

Lystavan et al. discovered a malonylated amaranthin (6′-O-malonyl-amaranthin) in a callus culture of *Celosia argentea* var. *cristata* and named the new compound celoscristatin [38]. The study did not describe the biosynthetic pathway, the specific enzymes involved, or the sequential order in which malonylation and glucuronidation supposedly occurred. We have recently identified a BAHD acyltransferase from red dragon fruit, *Hp*BAHD3, capable of malonylating betanin at carbon C6′, producing 6′-malonyl-betanin, commonly referred to as phyllocactin [39]. With the discovery of three GlcATs capable of producing amaranthin in *S. cerevisiae* and *Y. lipolytica*, we had both enzyme types required for celoscristatin formation. Thus, we sought to test whether the co-expression of the malonylating and glucuronidating enzymes in a betanin-producing platform strain could result in the production of celoscristatin (Fig. 6a). We created *S. cerevisiae* strains expressing the UDP-glucose dehydrogenase *At*UGD1 and *Hp*BAHD3 (the acyltransferase) in combination with one of the three GlcATs: *Ah*AmaSy (ST14125), *Cq*AmaSy1 (ST14126), or *Cc*AmaSy1 (ST14127). The phyllocactin-producing strain ST13946 (expressing *Hp*BAHD3) and the amaranthin-producing strain ST14115 (expressing *At*UGD1 and *Ah*AmaSy1) were included for reference.

In the HPLC analysis, a novel peak emerged in the strains co-expressing both decorating enzymes, with a RT of 6.4 min (Fig. 6b). The RT closely matched the reported RT for celoscristatin [38]. Additionally, all three engineered strains produced reduced levels of amaranthin and formed significantly reduced levels of phyllocactin compared to their respective parent strains. To confirm the identity of the new compound, the supernatants were analysed by LC-MS (Fig. S5a). MS and MS^2^ data confirmed the presence of celoscristatin (*m*/*z* = 813.1832). The highest titres of celoscristatin were observed in the strain expressing *Cq*AmaSy1, followed by *Cc*AmaSy1 (Fig. S5b). Since absolute quantification of celoscristatin is yet to be completed, peak areas were compared.

Afterwards, *Hp*BAHD3 was integrated into the *Yarrowia* strain expressing *Cc*AmaSy1. The resulting strain (ST14760) was cultivated on a small scale along with the amaranthin-producing strain ST14102 and the phyllocactin-producing strain ST14103. Total and extracellular production were analysed by HPLC (Fig. 6c). Like *S. cerevisiae*, the new *Yarrowia* strain produced a novel compound with an RT matching that of celoscristatin. LC-MS analysis confirmed that also in *Yarrowia*, co-expression of the acyltransferase and GlcAT led to the formation of celoscristatin (Fig. S5a). Like in *S. cerevisiae*, ST14760 produced negligible amounts of phyllocactin and amaranthin, instead favouring the production of celoscristatin. However, the total celoscristatin titre was lower than the titres of amaranthin and phyllocactin in the respective parent strains (Fig. 6d). What stood out was the significant difference between the extracellular and total fractions. While the extracellular fraction was orange-brown, the total fraction retained the deep pink-red hue, also characteristic of phyllocactin and amaranthin (Fig. 6e). The absorbance spectrum reflected this observation. The HPLC data confirmed that only 15 % of celoscristatin was secreted into the media, with the majority remaining inside the yeast cells. The *λ*_max_ of the total fraction from ST14760 was 537 nm, matching that of the amaranthin-producing strain.

That celoscristatin is produced by co-expression of *Hp*BAHD3 and each of the GlcATs illustrates the promiscuity of at least one of the two enzymes. Since the order of the biosynthesis pathway is unknown, it could either be that the GlcAT can glucuronidate phyllocactin instead of betanin (or cDOPA-malonylglucoside instead of cDOPA-glucoside) or that the BAHD AT can malonylate amaranthin instead of betanin (or cDOPA-glucuronosyl-glucoside instead of cDOPA-glucoside).

As ornamental plant, *Celosia argentea* does not typically find use in the food industry. Therefore, little is known about the properties of celoscristatin. For its use as food colourant, the pigment's stability and tinctorial strength would be of special interest. Unfortunately, the poor transport efficiency of celoscristatin, with only a small fraction being secreted into the medium, poses a significant challenge for its biotechnological production, hindering the scalability of celoscristatin production without further engineering or process adaptation.

Interestingly, we detected small amounts of bougainvillein-r I in ST14103 and ST12603 (Fig. S6a). Since no heterologous glycosyltransferase (glucosyltransferase or GlcAT) was integrated into the strains, an endogenous glycosyltransferase from *Y. lipolytica* must be able to synthesise the di-glucosylated betacyanin (betanidin 5-O-β-sophoroside). While screening for new betalain variants using LC-MS, we discovered that the phyllocactin-producing ST14103 produced small amounts of a compound with an *m*/*z* of 799.2040, corresponding to mammillarinin (Fig. S6b). Mammillarinin (6′-O-malonyl-bougainvillein-r I) is a malonylated derivative of bougainvillein-r I and has been identified as one of the dominant betacyanins in several species of the *Mammillaria* genus [37]. Beyond this, little is known about the compound, except that it has a reported *λ*_max_ of 539 nm. In our yeast strains, mammillarinin is produced in small quantities, rendering it irrelevant for industrial applications at this stage.

## 4 Conclusion

In this study, we have shown that not only betanin, but also several betanin derivatives, namely bougainvillein-r I, amaranthin and celoscristatin, can heterologously be produced in microbial hosts. Three glucuronosyltransferases from different betalain-producing plants were screened in *S. cerevisiae* and then transferred to a *Y. lipolytica* betanin-producer strain. Without additional engineering, almost all betanin was glucuronidated to amaranthin. Co-expression of a glucuronosyltransferase with a malonyltransferase led to the formation of malonylated amaranthin. This work illustrates the suitability of *Y. lipolytica* for the gram-scale production of betalains and demonstrates the potential of combining decorating enzymes to further expand the betalain portfolio for their application as food colourants.

## CRediT authorship contribution statement

**Christiane Glitz:** Writing – review & editing, Writing – original draft, Visualization, Investigation, Conceptualization. **Jane Dannow Dyekjær:** Software, Methodology, Investigation. **Gian Maria Cristian Solimando:** Investigation. **Paulo Marcelo Avila Neto:** Investigation. **Daniela Rago:** Methodology, Investigation, Formal analysis. **Mahsa Babaei:** Supervision, Conceptualization. **Irina Borodina:** Supervision, Funding acquisition, Conceptualization.

## Declaration of generative AI in scientific writing

During the preparation of this work the authors used ChatGPT in order to improve language, readability and grammar. After using this tool, the authors reviewed and edited the content as needed and take full responsibility for the content of the publication.

## Funding

This work was supported by funding from the 10.13039/501100009708Novo Nordisk Foundation (grant agreement numbers NNF21OC0072559, NNF20CC0035580 and NNF20OC0060809) and from the 10.13039/501100000781European Research Council under the 10.13039/501100007601European Union's Horizon 2020 research and innovation programme (grant agreement number 101123257).

## Declaration of competing interest

The authors declare the following financial interests/personal relationships which may be considered as potential competing interests: IB, MB, JDD and CG are co-inventors on a patent application related to this research. The remaining authors declare no competing interests.
