# Supplementary material for: Recombinant production of amaranthin and other betalain variants with yeast cell factories
Source: Synth Syst Biotechnol. 2025 May 21;10(4):1127–39. doi: 10.1016/j.synbio.2025.05.008 (PMC12221589; doi:10.1016/j.synbio.2025.05.008)
Supplement: Multimedia component 1 [file mmc1.docx]

**Supporting Information 1**

**Recombinant production of amaranthin and other betalain variants with yeast cell factories**

1. **Supporting Figures and Tables**
2. **Biological Materials**
3. **References**
4. **Supporting Figures and Tables**


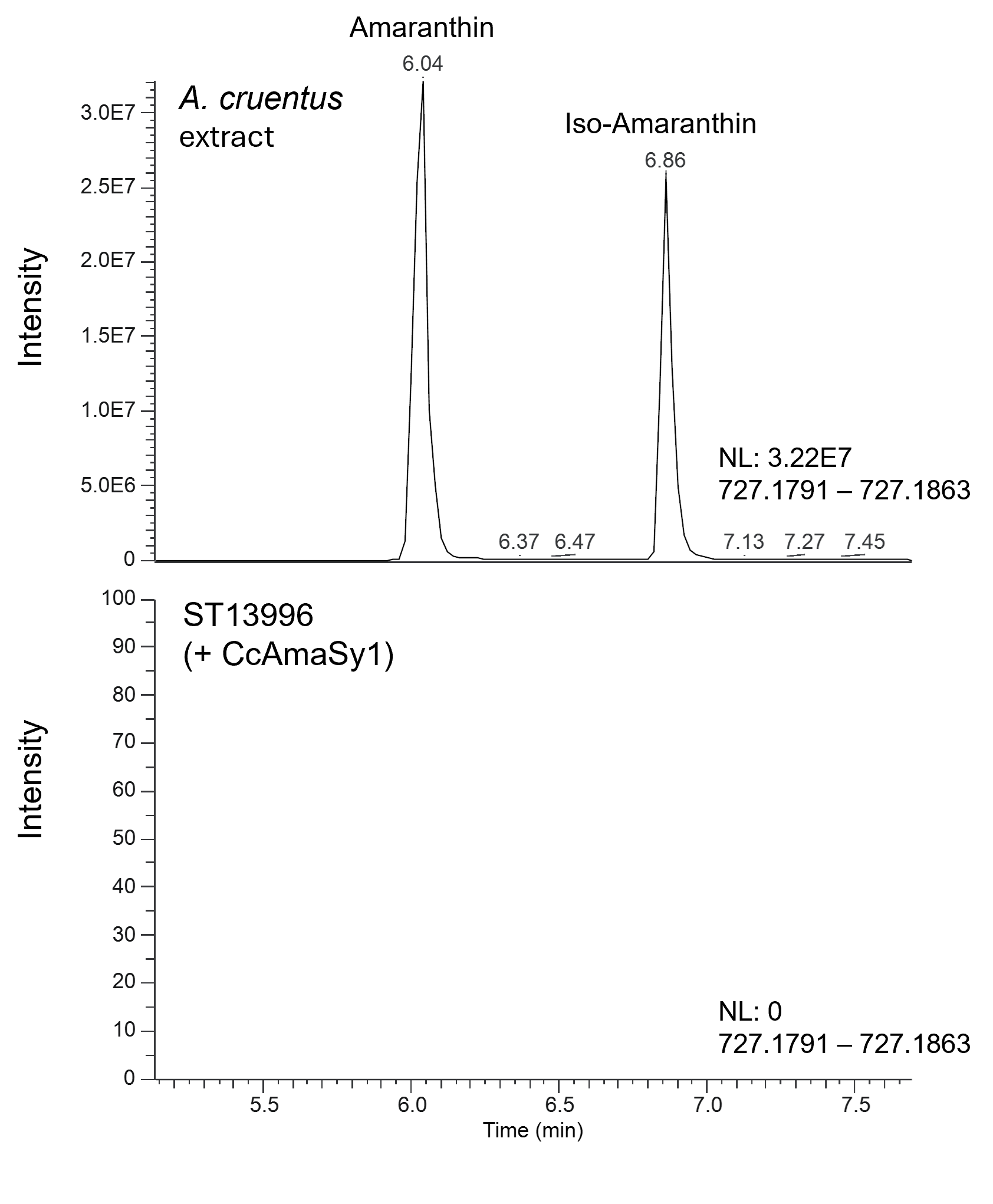


**Figure S1. LC-MS analysis of *Cc*AmaSy1-expressing *S. cerevisiae* strain**. Extracted-ion chromatogram (XIC) of amaranthin (m/z 727.1829) and its isoform from *A. cruentus* plant extract together with *S. cerevisiae* strain ST13996 (↑*Cc*AmaSy1). While the plant contains amaranthin and isoamaranthin, neither was detected in the yeast strain.


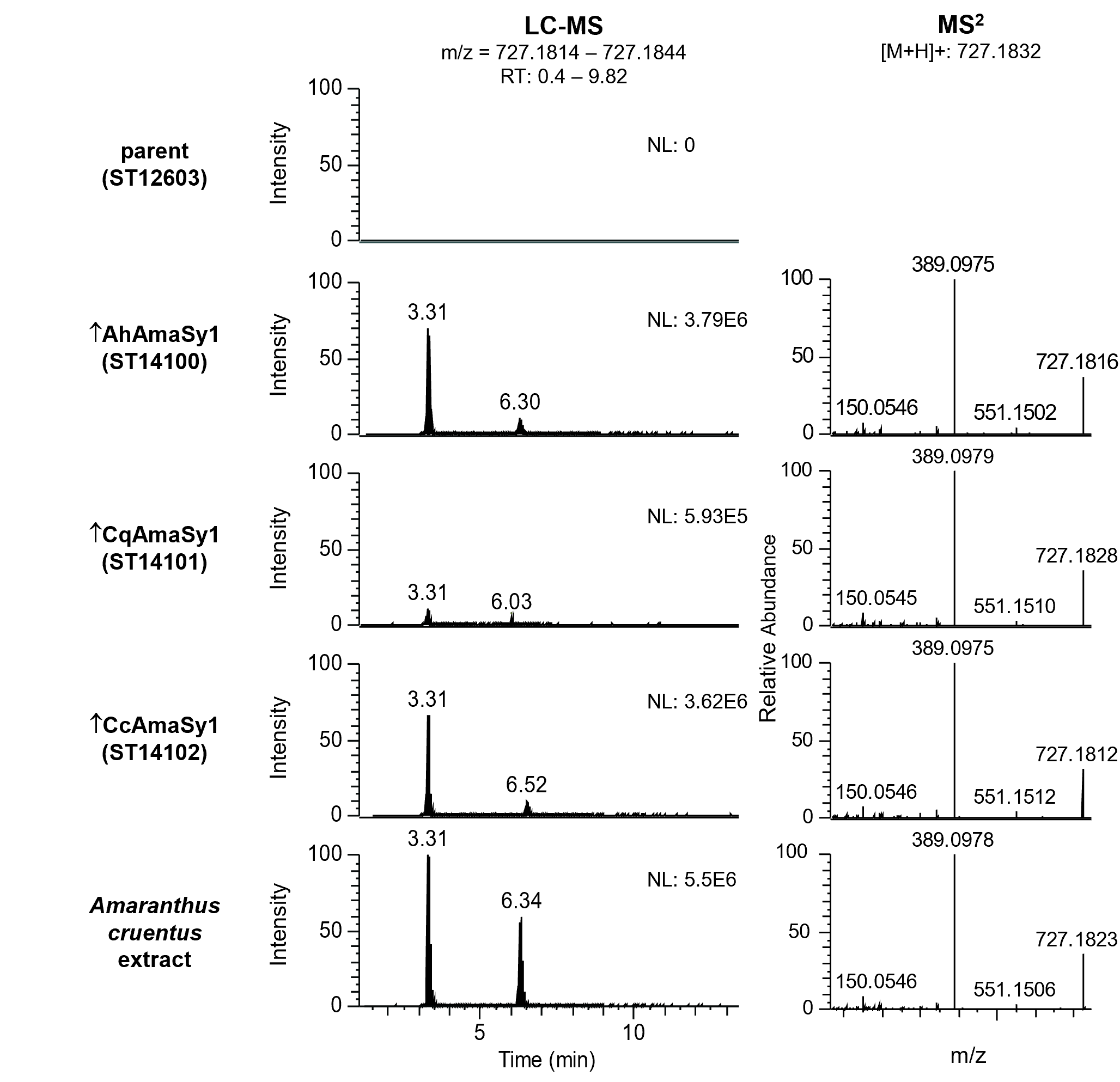


**Figure S2**. **LC-MS analysis of glucuronosyltransferase-expressing *Y. lipolytica*.** XIC of amaranthin (m/z 727.1829) and the corresponding MS^2^ spectrum from the LC-MS analysis of *Y. lipolytica* strains expressing a glucuronosyltransferase in the betanin-producing parent strain ST12603 (total fraction). All three strains mainly produce amaranthin and small amounts of isoamaranthin. The upward arrow (↑) indicates overexpression of the gene listed after the symbol.

**
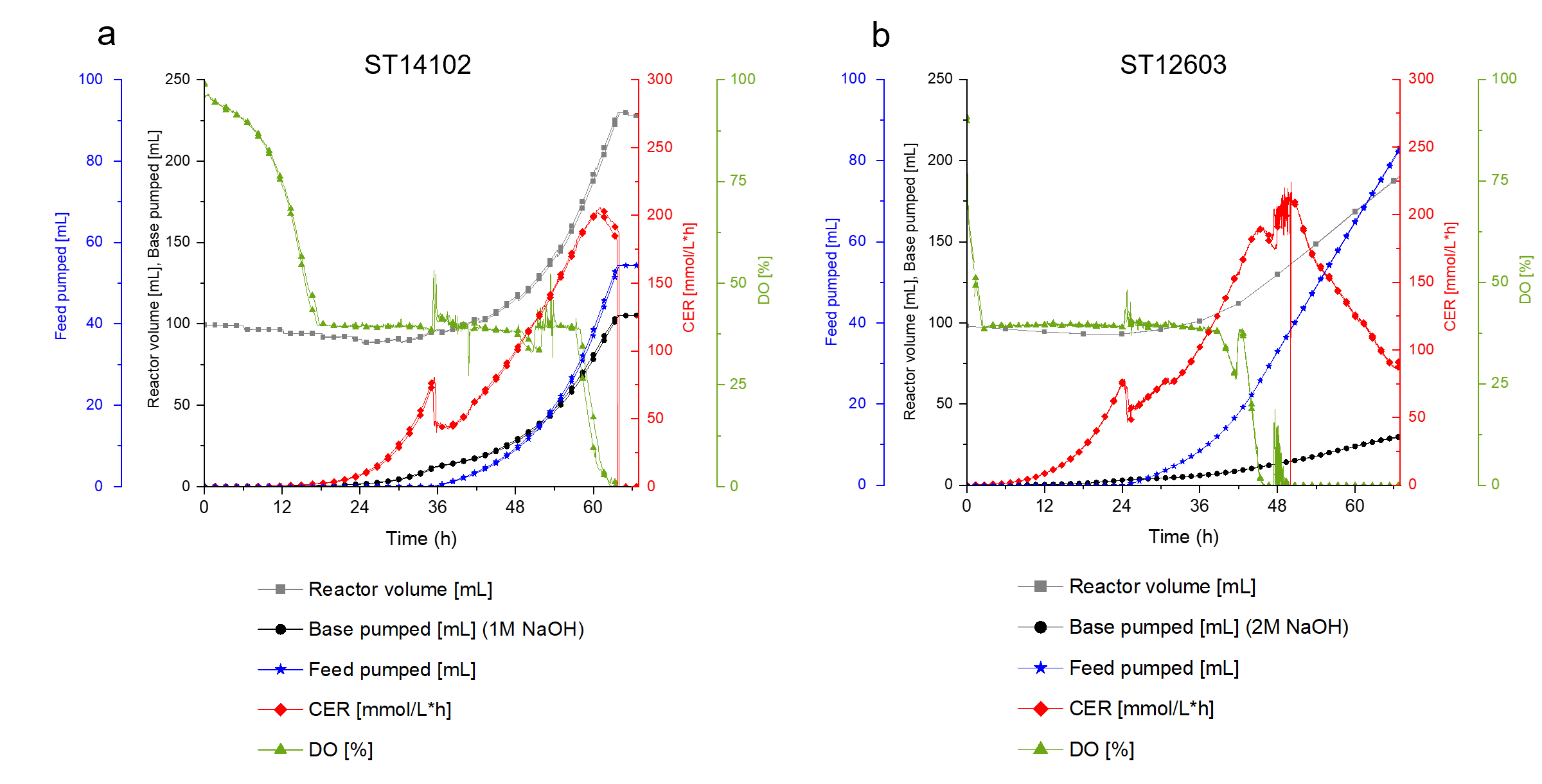
**

**Figure S3**. **Process data of fed-batch fermentations.** **a** Amaranthin-producing *Y. lipolytica* ST14102 **b** Betanin-producing ST12603. For each strain, the data of both replicates is plotted.


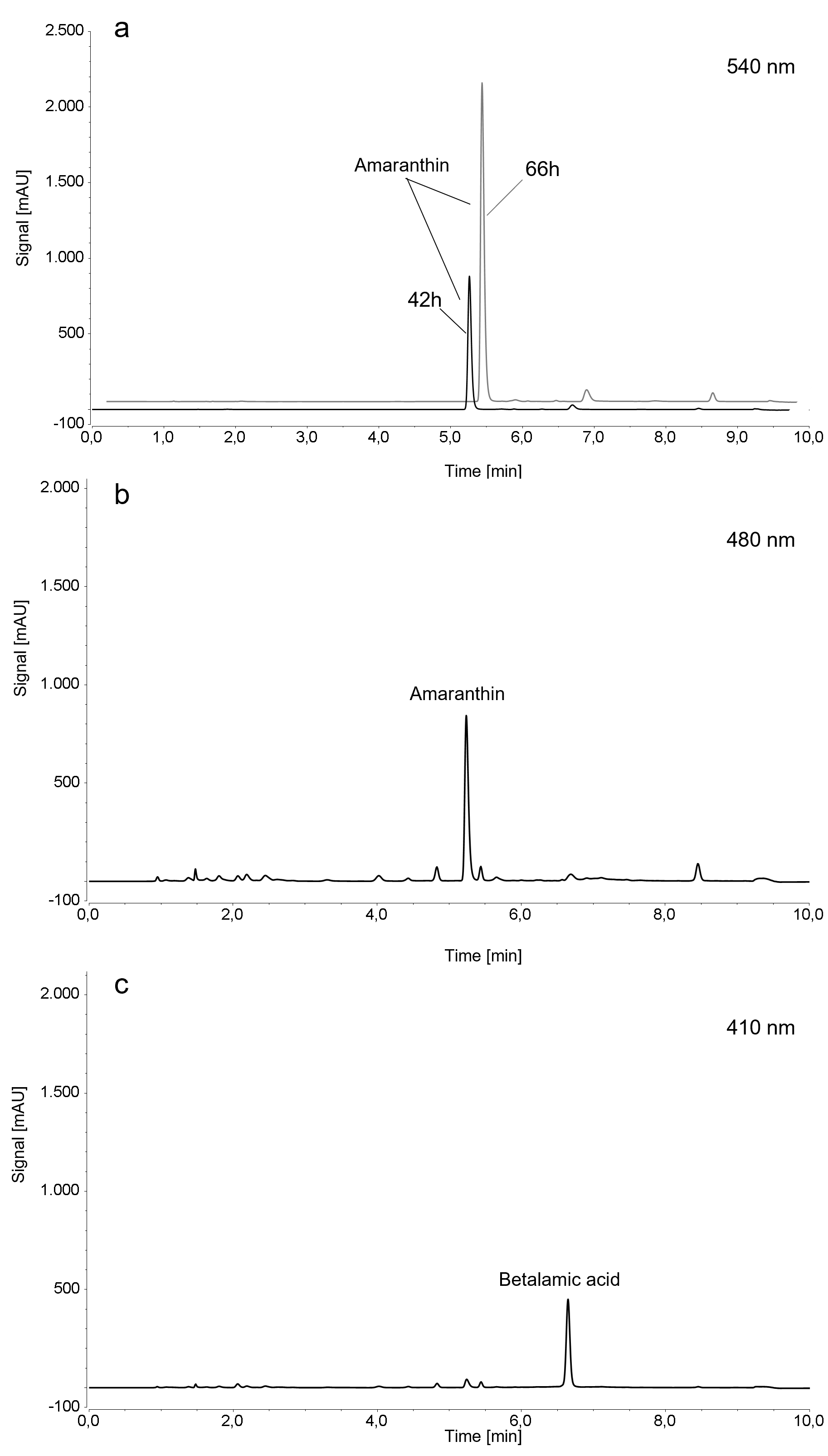


**Figure S4.** **HPLC chromatograms of 10x diluted samples of ST14102 in fed-batch fermentation**. **a** Profile at 540 nm, corresponding to betacyanins. Samples after 42 h and 66 h fermentation. **b** Profile at 480 nm, corresponding to betaxanthins but also showing the shoulders of the amaranthin peak. **c** Profile at 410 nm, characteristic for betalamic acid.


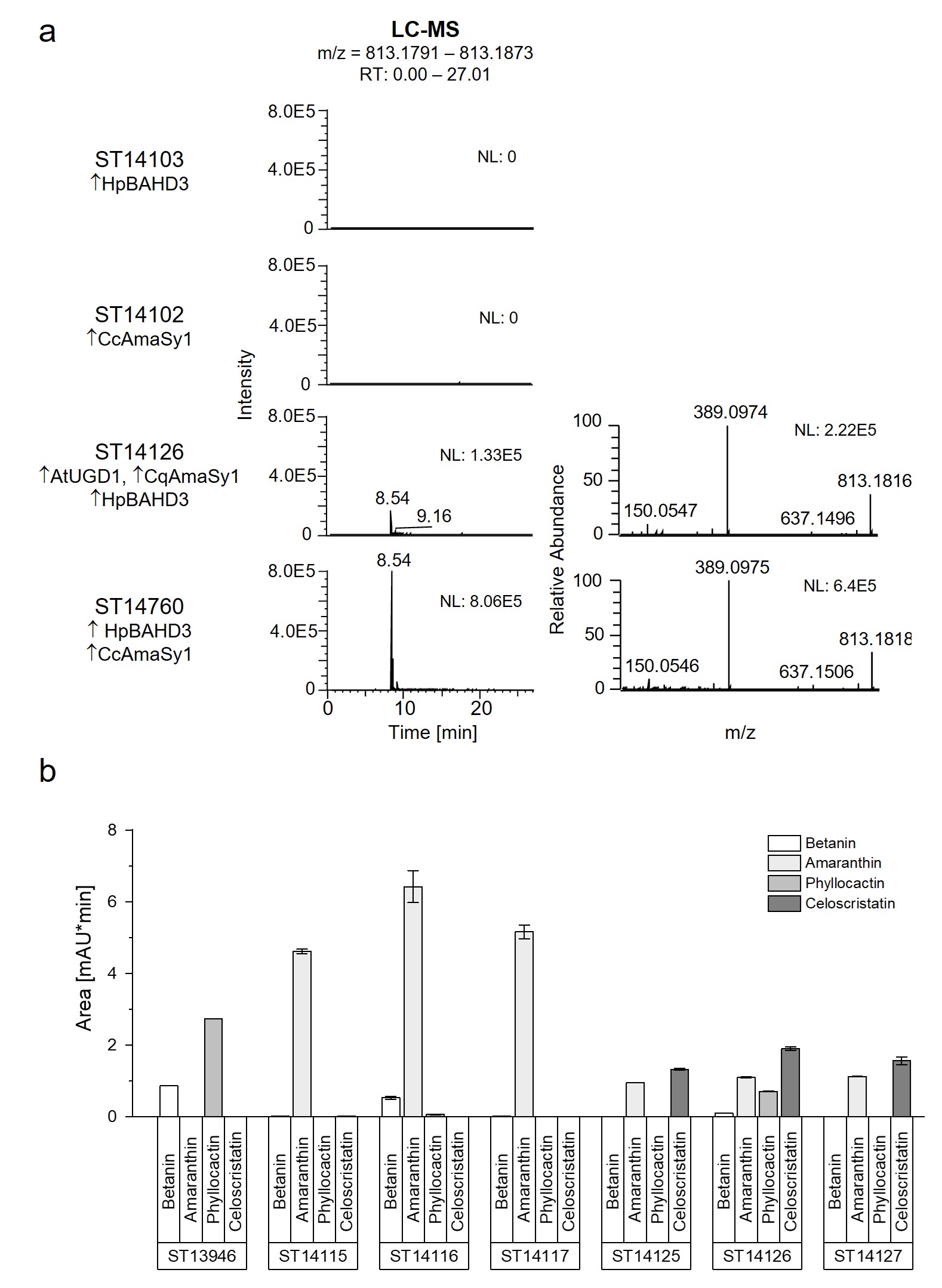


**Figure S5.** **Celoscristatin formation in *S. cerevisiae* and *Y. lipolytica*. a** A compound with m/z 813.1832 was detected in *S. cerevisiae* strain ST14126 and *Y. lipolytica* strain ST14760 but absent in the strains expressing only the acyltransferase (ST14103) or one glucuronosyltransferase (ST14102). The MS^2^ fragmentation pattern matches celoscristatin. **b** Betalain production in *S. cerevisiae* strains. Peak areas (mAU*min) are compared. The upward arrow (↑) indicates overexpression of the gene listed after the symbol.


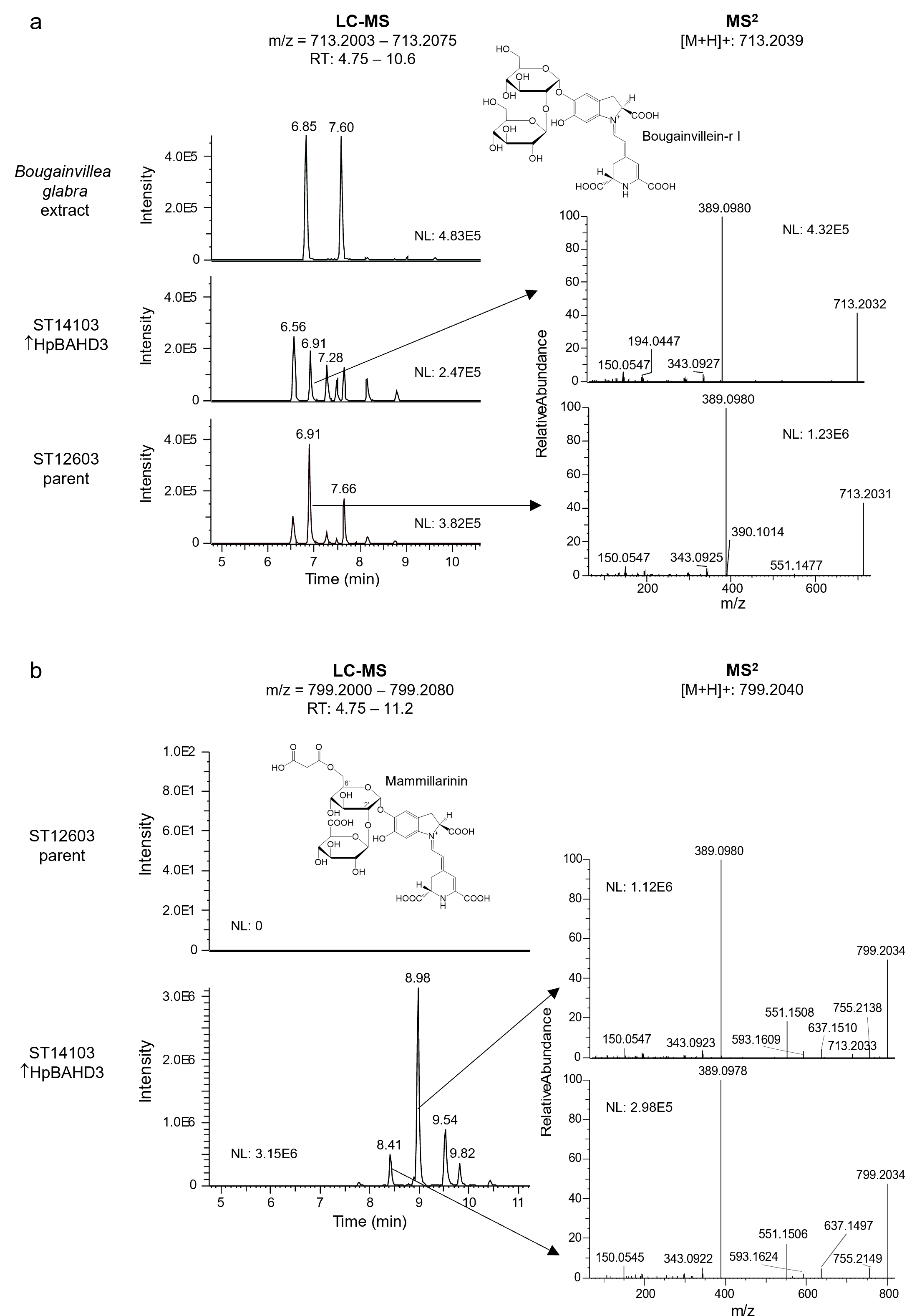


**Figure S6**. **LC-MS analysis of *Y. lipolytica* strains ST12603 and ST14103 (↑*Hp*BAHD3).** **a** In both strains, bougainvillein-r I (XIC of m/z = 713.2039) was detected. **b** Expression of the acyltransferase *Hp*BAHD3 from *H. polyrhizus* led to the formation of mammillarinin, malonylated bougainvillein-r I (XIC of m/z = 799.2040) in strain ST14103. The MS^2^ spectra of the two peaks at 8.41 min and at 8.98 min indicate that mammillarinin (6’-O-malonyl-bougainvillein-r I) and isomammillarinin (6’-O-malonyl-isobougainvillein-r I) were formed. The remaining peaks at 9.54 min and 9.82 min could possibly correspond to 4’-O-malonyl-bougainvillein-r I and 4’-O-malonyl-isobougainvillein-r I, however, their MS^2^ spectra were not sufficiently conclusive. The upward arrow (↑) indicates overexpression of the gene listed after the symbol.

**Table S1.** Raw data for the calculation of calibration curves for the described betacyanin variants. From the absorbance values of pure compounds (commercial standards or purified from plant extracts) and their area (mAU*min) in the HPLC, the concentration of the samples was determined and used to calculate a calibration curve for each compound.

| **Compound** | **e (L/cm*mol)** | **MW (g/mol)** | **mg/L** | **umol/L** | **mAU*min** | **Slope m (b = 0)** |
| --- | --- | --- | --- | --- | --- | --- |
| **Betanin** | 65000 | 550 |  |  |  | **0.703** |
| Betanin_1 |  |  | 0.41 | 0.74 | 0.27 |  |
| Betanin_2 |  |  | 0.81 | 1.47 | 0.54 |  |
| Betanin_3 |  |  | 1.59 | 2.89 | 1.09 |  |
| Betanin_4 |  |  | 3.91 | 7.11 | 2.63 |  |
| Betanin_5 |  |  | 7.77 | 14.13 | 5.53 |  |
| Betanin 6 |  |  | 15.50 | 28.18 | 10.9 |  |
| **Isobetanin** | 65000 | 550 |  |  |  | **0.719** |
| Isobetanin_1 |  |  | 0.41 | 0.74 | 0.28 |  |
| Isobetanin_2 |  |  | 0.81 | 1.47 | 0.56 |  |
| Isobetanin_3 |  |  | 1.59 | 2.89 | 1.12 |  |
| Isobetanin_4 |  |  | 3.91 | 7.11 | 2.68 |  |
| Isobetanin_5 |  |  | 7.77 | 14.13 | 5.66 |  |
| Isobetanin 6 |  |  | 15.50 | 28.18 | 11.15 |  |
| **Amaranthin** | 65000 | 726.6 |  |  |  | **0.421** |
| Amaranthin 0.1% |  |  | 0.12 | 0.17 | 0.12 |  |
| Amaranthin 1% |  |  | 0.97 | 1.34 | 0.47 |  |
| Amaranthin 10% |  |  | 9.61 | 13.23 | 4.65 |  |
| Amaranthin 25% |  |  | 23.03 | 31.69 | 11.29 |  |
| Amaranthin 50% |  |  | 46.17 | 63.54 | 22.37 |  |
| Amaranthin 100% |  |  | 99.91 | 137.51 | 44.83 |  |
| Amaranthin 500% |  |  | 499.57 | 687.54 | 209.39 |  |
| **Bougainvillein-r I** | 65000 | 712.6 |  |  |  | **0.286** |
| Bougainvillein-r I_25% |  |  | 0.15 | 0.21 | 0.04 |  |
| Bougainvillein-r I_50% |  |  | 0.30 | 0.42 | 0.08 |  |
| Bougainvillein-r I 100% |  |  | 0.60 | 0.84 | 0.17 |  |
| **Phyllocactin** | 65000 | 637.5 |  |  |  | **0.600** |
| Phyllocactin 1% |  |  | 2.37 | 3.72 | 1.81 |  |
| Phyllocactin 10% |  |  | 23.73 | 37.23 | 13.55 |  |
| Phyllocactin 25% |  |  | 55.90 | 87.69 | 33.05 |  |
| Phyllocactin 50% |  |  | 109.85 | 172.31 | 67.75 |  |
| Phyllocactin 100% |  |  | 237.35 | 372.31 | 141.81 |  |

**Table S2**. Summary of the compounds detected and quantified in this manuscript by HPLC-UV and LC-MS. Max. absorbance differs between sources.

| **Compound** | **Trivial name** | **Molecular weight [g/mol]** | **λ_max_ [nm]** | **Retention time (HPLC) [min]** | **m/z [M+H]^+^** | **MS^2^ (expected)** |
| --- | --- | --- | --- | --- | --- | --- |
| Betanidin | Betanidin | 388.3 | 545 [1] | 6.5 | 389.0984 | 325; 297; 255; 241; 149 |
| Betanidin 5-O-β-glucoside | Betanin | 550.5 | 535 [1,2] | 5.7 | 551.1513 | 389; 343; 150 |
| Betanidin 5-O-β-glucoside | Isobetanin | 550.5 | 535 [1,2] | 6.15 | 551.1513 | 389; 343; 150 |
| Betalamic acid | Betalamic acid | 211.17 | 410 [3] | 6.68 |  |  |
| 6’-O-Malonyl-betanin | Phyllocactin (I) | 637.5 | 533 [1]  537 [2]  539 [4] | 6.7 | 637.1517 | 619; 593; 551; 389; 343; 150 |
| 6’-O-Malonyl-isobetanin | Isophyllocactin (I) | 637.5 | 533 [1]  537 [2]  538 [4] | 7.12 | 637.1517 | 619; 593; 551; 389; 343; 150 |
| Betanidin 5-O-β-sophoroside | Bougainvillein-r I  (Melocactin) | 712.6 | 538 [5]  540 [6]  534 [1] |  | 713.2039 | 551; 389; 343; 150 |
| Isobetanidin 5-O-β-sophoroside | Isobougainvillein-r I  (Isomelocactin) | 712.6 | 534 [1] |  | 713.2039 | 551; 389; 343; 150 |
| Betanidin 5-O-(2’-O- β -glucuronosyl)-glucoside | Amaranthin | 726.6 | 536 [1,7] |  | 727.1829 | 551; 389; 343; 150 |
| Isobetanidin 5-O-(2’-O- β -glucuronosyl)-glucoside | Isoamaranthin | 726.6 | 536 [7]  539 [1] |  | 727.1829 | 551; 389; 343; 150 |
| 6’-O-malonyl-amaranthin | Celoscristatin | 812.6 | 536 [7] |  | 813.1832 | 795; 769; 727; 637; 593; 551; 389; 343; 150 |
| 6’-O-malonyl-bougainvillein-r I | Mammillarinin | 798.7 | 539 [5] | Not identified | 799.2040 | 755; 713; 637; 551; 389; 343; 150 |

1. **Biological Materials**

**Table S3**. Strains used in this study

| **Strain**  **name** | **Genotype** | **Parent**  **strain** | **Repair vector** | **Reference** |
| --- | --- | --- | --- | --- |
| ***S. cerevisiae*** | | | | |
| ST12160 | CEN.PK113-7D + pCas9-KanMX  X-2::tADH1-MjDOD-pTEF1-pPGK1-TYH^W13L^-tCYC1  X-3::pTEF1-BvGT2-tADH1 | ST9942 | See ref. | [8] |
| ST13931 | CEN.PK113-7D + pCas9-KanMX  X-2::tADH1-MjDOD-pTEF1-pPGK1-TYH^W13L^-tCYC1  X-3::pTEF1-BvGT2-tADH1  XII-5::tADH1-AhAmaSy1-pTEF1 | ST12160 | pCfB12307 | This study |
| ST13932 | CEN.PK113-7D + pCas9-KanMX  X-2::tADH1-MjDOD-pTEF1-pPGK1-TYH^W13L^-tCYC1  X-3::pTEF1-BvGT2-tADH1  XII-5::tADH1-CqAmaSy1-pTEF1 | ST12160 | pCfB12308 | This study |
| ST13946 | CEN.PK113-7D + pCas9-KanMX  X-2::tADH1-MjDOD-pTEF1-pPGK1-TYH^W13L^-tCYC1  X-3::pTEF1-BvGT2-tADH1  XI-5::tADH1-HpBAHD3-pTEF1 | ST12160 | pCfB12303 | [9] |
| ST13996 | CEN.PK113-7D + pCas9-KanMX  X-2::tADH1-MjDOD-pTEF1-pPGK1-TYH^W13L^-tCYC1  X-3::pTEF1-BvGT2-tADH1  XII-5::tADH1-CcAmaSy1-pTEF1 | ST12160 | pCfB12309 | This study |
| ST14115 | CEN.PK113-7D + pCas9-KanMX  X-2::tADH1-MjDOD-pTEF1-pPGK1-TYH^W13L^-tCYC1  X-3::pTEF1-BvGT2-tADH1  XII-5::tADH1-AhAmaSy1-pTEF1  XI-2::tADH1-AtUGD1-pTEF1 | ST13931 | pCfB12413 | This study |
| ST14116 | CEN.PK113-7D + pCas9-KanMX  X-2::tADH1-MjDOD-pTEF1-pPGK1-TYH^W13L^-tCYC1  X-3::pTEF1-BvGT2-tADH1  XII-5::tADH1-CqAmaSy1-pTEF1  XI-2::tADH1-AtUGD1-pTEF1 | ST13932 | pCfB12413 | This study |
| ST14117 | CEN.PK113-7D + pCas9-KanMX  X-2::tADH1-MjDOD-pTEF1-pPGK1-TYH^W13L^-tCYC1  X-3::pTEF1-BvGT2-tADH1  XII-5::tADH1-CcAmaSy1-pTEF1  XI-2::tADH1-AtUGD1-pTEF1 | ST13996 | pCfB12413 | This study |
| ST14118 | CEN.PK113-7D + pCas9-KanMX  X-2::tADH1-MjDOD-pTEF1-pPGK1-TYH^W13L^-tCYC1  X-3::pTEF1-BvGT2-tADH1  XI-2::tADH1-AtUGD1-pTEF1 | ST12160 | pCfB12413 | This study |
| ST14125 | CEN.PK113-7D + pCas9-KanMX  X-2::tADH1-MjDOD-pTEF1-pPGK1-TYH^W13L^-tCYC1  X-3::pTEF1-BvGT2-tADH1  XII-5::tADH1-AhAmaSy1-pTEF1  XI-2::tADH1-AtUGD1-pTEF1  XI-5::tADH1-HpBAHD3-pTEF1 | ST14115 | pCfB12303 | This study |
| ST14126 | CEN.PK113-7D + pCas9-KanMX  X-2::tADH1-MjDOD-pTEF1-pPGK1-TYH^W13L^-tCYC1  X-3::pTEF1-BvGT2-tADH1  XII-5::tADH1-CqAmaSy1-pTEF1  XI-2::tADH1-AtUGD1-pTEF1  XI-5::tADH1-HpBAHD3-pTEF1 | ST14116 | pCfB12303 | This study |
| ST14127 | CEN.PK113-7D + pCas9-KanMX  X-2::tADH1-MjDOD-pTEF1-pPGK1-TYH^W13L^-tCYC1  X-3::pTEF1-BvGT2-tADH1  XII-5::tADH1-CcAmaSy1-pTEF1  XI-2::tADH1-AtUGD1-pTEF1  XI-5::tADH1-HpBAHD3-pTEF1 | ST14117 | pCfB12303 | This study |
| ***Yarrowia lipolytica*** | | | | |
| ST12603 | MATA ku70∆::pTEF1-Cas9-tTEF12::pGPD-DsdA-tLIP2  IntE1::tPex20-YlARO7^G139S^-pGPD-pTEFin-ARO4^K221L^-tLIP2  IntE3::tPEX20-BvGT2-pTEFin-tPEX20-MjDOD-pGPD-pTEFin-EvTYH-tLIP2  IntE4::tPEX20-BvGT2-pTEFin-tPEX20-MjDOD-pGPD-pTEFin-EvTYH-tLIP2  IntF3::tPEX20-BvGT2-pTEFin-tPEX20-MjDOD-pGPD-pTEFin-EvTYH-tLIP2  ∆4hppd |  |  | see ST12376 [10] |
| ST14100 | MATA ku70∆::pTEF1-Cas9-tTEF12::pGPD-DsdA-tLIP2  IntE1::tPex20-YlARO7^G139S^-pGPD-pTEFin-ARO4^K221L^-tLIP2  IntE3::tPEX20-BvGT2-pTEFin-tPEX20-MjDOD-pGPD-pTEFin-EvTYH-tLIP2  IntE4::tPEX20-BvGT2-pTEFin-tPEX20-MjDOD-pGPD-pTEFin-EvTYH-tLIP2  IntF3::tPEX20-BvGT2-pTEFin-tPEX20-MjDOD-pGPD-pTEFin-EvTYH-tLIP2  ∆4hppd  IntC3::pGPD-AhAmaSy1-tLIP2 | ST12603 | pCfB12418 | This study |
| ST14101 | MATA ku70∆::pTEF1-Cas9-tTEF12::pGPD-DsdA-tLIP2  IntE1::tPex20-YlARO7^G139S^-pGPD-pTEFin-ARO4^K221L^-tLIP2  IntE3::tPEX20-BvGT2-pTEFin-tPEX20-MjDOD-pGPD-pTEFin-EvTYH-tLIP2  IntE4::tPEX20-BvGT2-pTEFin-tPEX20-MjDOD-pGPD-pTEFin-EvTYH-tLIP2  IntF3::tPEX20-BvGT2-pTEFin-tPEX20-MjDOD-pGPD-pTEFin-EvTYH-tLIP2  ∆4hppd  IntC3::pGPD-CqAmaSy1-tLIP2 | ST12603 | pCfB12420 | This study |
| ST14102 | MATA ku70∆::pTEF1-Cas9-tTEF12::pGPD-DsdA-tLIP2  IntE1::tPex20-YlARO7^G139S^-pGPD-pTEFin-ARO4^K221L^-tLIP2  IntE3::tPEX20-BvGT2-pTEFin-tPEX20-MjDOD-pGPD-pTEFin-EvTYH-tLIP2  IntE4::tPEX20-BvGT2-pTEFin-tPEX20-MjDOD-pGPD-pTEFin-EvTYH-tLIP2  IntF3::tPEX20-BvGT2-pTEFin-tPEX20-MjDOD-pGPD-pTEFin-EvTYH-tLIP2  ∆4hppd  IntC3::pGPD-CcAmaSy1-tLIP2 | ST12603 | pCFB12422 | This study |
| ST14103 | MATA ku70∆::pTEF1-Cas9-tTEF12::pGPD-DsdA-tLIP2  IntE1::tPex20-YlARO7^G139S^-pGPD-pTEFin-ARO4^K221L^-tLIP2  IntE3::tPEX20-BvGT2-pTEFin-tPEX20-MjDOD-pGPD-pTEFin-EvTYH-tLIP2  IntE4::tPEX20-BvGT2-pTEFin-tPEX20-MjDOD-pGPD-pTEFin-EvTYH-tLIP2  IntF3::tPEX20-BvGT2-pTEFin-tPEX20-MjDOD-pGPD-pTEFin-EvTYH-tLIP2  ∆4hppd  IntD1::pTEFin-HpBAHD3-tLIP2 | ST12603 | pCfB12430 |  |
| ST14760 | MATA ku70∆::pTEF1-Cas9-tTEF12::pGPD-DsdA-tLIP2  IntE1::tPex20-YlARO7^G139S^-pGPD-pTEFin-ARO4^K221L^-tLIP2  IntE3::tPEX20-BvGT2-pTEFin-tPEX20-MjDOD-pGPD-pTEFin-EvTYH-tLIP2  IntE4::tPEX20-BvGT2-pTEFin-tPEX20-MjDOD-pGPD-pTEFin-EvTYH-tLIP2  IntF3::tPEX20-BvGT2-pTEFin-tPEX20-MjDOD-pGPD-pTEFin-EvTYH-tLIP2  ∆4hppd  IntD1::pTEFin-HpBAHD3-tLIP2  IntC3::pGPD-CcAmaSy1-tLIP2 | ST14103 | pCfB12422 | This study |

**Table S4**. Plasmids used in this study

| **Plasmid** | **BioBricks** | **Characteristics** | **Reference** |
| --- | --- | --- | --- |
| **gRNA vector** | | | |
| pCfB3044 (XI-2) | -. | NAT | [11] |
| pCfB3046 (XI-5) | - | NAT | [11] |
| pCfB3050 (XII-5) | - | NAT | [12] |
| pCfB6630 (IntC3) | - | pNat-YLgRNA3-IntC3 | [12] |
| pCfB6631 (IntD1) | - | pNat-YLgRNA2-IntD1 | [12] |
|  |  |  |  |
| **Yeast genome integration vectors** | | | |
| pCfB12303 | BB3038, BB8, BB6564 | XI-5::tADH1-HpBAHD3-pTEF1 | [9] |
| pCfB12307 | BB3034, BB8, BB6571 | XII-5::tADH1-AhAmaSy1-pTEF1 | This study |
| pCfB12308 | BB3034, BB8, BB6572 | XII-5::tADH1-CqAmaSy1-pTEF1 | This study |
| pCfB12309 | BB3034, BB8, BB6573 | XII-5::tADH1-CcAmaSy1-pTEF1 | This study |
| pCfB12413 | BB3032, BB8, BB6700 | XI-2::tADH1-AtUGD1-pTEF1 | This study |
| pCfB12418 | BB5950, BB6739, BB6732 | IntC3::pGPD-AhAmaSy1-tLIP2 | This study |
| pCfB12420 | BB5950, BB6739, BB6733 | IntC3::pGPD-CqAmaSy1-tLIP2 | This study |
| pCfB12422 | BB5950, BB6739, BB6734 | IntC3::pGPD-CcAmaSy1-tLIP2 | This study |

**Table S5.** Biobricks used in this study

| **BioBrick** | **Primer fwd** | **Primer rev** | **Template** | **Description** |
| --- | --- | --- | --- | --- |
| BB008 | PR-1750 | PR-6 | CEN.PK113-5D genome | pTEF1 promoter from *S. cerevisiae* with USER overhangs for P1 |
| BB3032 | PR-22420 | PR-22421 | pCfB2903[11] | Backbone for XI-2 integration site with USER overhangs |
| BB3034 | PR-22420 | PR-22421 | pCfB2909[11] | Backbone for XII-5 integration site with USER overhangs |
| BB3038 | PR-22420 | PR-22421 | pCfB3037[11] | Backbone for XI-5 integration site with USER overhangs |
| BB5950 | PR-23968 | PR-23963 | pCfB6371[12] | Backbone for IntC3 integration site with USER overhangs |
| BB6571 | PR-32630 | PR-32631 | Synthetic gene string | AhAmaSy1 with USER overhangs for G1 |
| BB6572 | PR-32632 | PR-32633 | Synthetic gene string | CqAmaSy1 with USER overhangs for G1 |
| BB6573 | PR-32634 | PR-32635 | Synthetic gene string | CcAmaSy1 with USER overhangs for G1 |
| BB6700 | PR-33125 | PR-33126 | Synthetic gene string | AtUGD1 with USER overhangs for G1 |
| BB6732 | PR-33146 | PR-33147 | Synthetic gene string | AhAmaSy with USER overhangs for G1 (*Y. lipolytica*) |
| BB6733 | PR-33150 | PR-33151 | Synthetic gene string | CqAmaSy with USER overhangs for G1 in (*Y. lipolytica*) |
| BB6734 | PR-33154 | PR-33155 | Synthetic gene string | CcAmaSy with USER overhangs for G1 in (*Y. lipolytica*) |
| BB6739 | PR-23965 | PR-33238 | W29 genome | pGPD promoter from *Y. lipolytica* with USER overhangs for P1 |

**Table S6**. Primers used in this study

| **Primer** | **Sequence (5’ → 3’)** |
| --- | --- |
| PR-6 | CACGCGAUGCACACACCATAGCTTC |
| PR-1750 | ACCTGCACUTTGTAATTAAAACTTAGATTAGATTG |
| PR-22420 | ATCGCACGUGTAGATACGTTGTTGACACTTC |
| PR-22421 | ATCGCGTGUATCCGCTCTAACCGAAAAGGAAG |
| PR-23963 | ATCGCACGUAAGTGTGGATGGGGAAGTGAGT |
| PR-23965 | ACCTGCACUTGTTGATGTGTGTTTAATTCAAGAATGAAT |
| PR-23968 | ATCGCGTGUCTTCTGTTCGGAATCAACCTCAAGG |
| PR-32630 | CGTGCGAUTCACACGATCAATAATTGCTG |
| PR-32631 | AGTGCAGGUAAAACAATGAGCCACAATAAAG |
| PR-32632 | CGTGCGAUCTAAGATCCGATCAGCTG |
| PR-32633 | AGTGCAGGUAAAACAATGTCACAGAACAAGG |
| PR-32634 | CGTGCGAUTCATCCAAGTAATTGC |
| PR-32635 | AGTGCAGGUAAAACAATGAGTTCTAATCAGG |
| PR-33125 | CGTGCGAUTCAAGCCACTGCAGGC |
| PR-33126 | AGTGCAGGUAAAACAATGGTAAAGATTTGCTG |
| PR-33146 | ACGTGCGAUTCAGACGATGAGGAGTTGC |
| PR-33147 | AGTGCAGGUGCCACAATGTCCCACAACAAGGAGTCG |
| PR-33150 | ACGTGCGAUTTAGCTTCCAATCAGC |
| PR-33151 | AGTGCAGGUGCCACAATGTCCCAAAATAAAGAC |
| PR-33154 | ACGTGCGAUCTAGCCAAGCAGCTGTCTG |
| PR-33155 | AGTGCAGGUGCCACAATGTCGTCCAACCAGGAC |
| PR-33238 | ACACGCGAUGACGCAGTAGGATGTCC |

**Table S7.** Genes used in this study

| **Gene** | **Sequence** |
| --- | --- |
| ***Hp*BAHD3**  [9] | **Amino acid sequence** |
|  | MVNQEGIKVISECFVRPEHEVEAAKNPLSLGPVDLAFLSIDPIQKGLLFPFVTNDTRPEISSIVERLKRSLSLALVYFYPLAGRFETNKYEDEHACWIFLDCTKGPGARLIHASYEDLSVSDILSSTDVHPAVRSFFDLGERVVNYDGHTKALLSIQVTELLDGVFIGFSMNHSVVDGTSFIHFVSSLSEIFRSDPQGNEGPVKISRVPLYKMHAPEGCGPISKLPYLEHDEFIVRYDPGPLRERIFHFSPESMARLKAKANEECGSGTQEISSYMALSSLVWKSITRAYNPAGHDETCCFTALNARPRHNPPLSDDYFGNILVRAKGVSKVDELLSQSLGWGARILSQEVKKHTHETICETLKMFAEHPSVIRRGLNQPGFYSVDNGGSHRFDMYGPEFGLGRAVAVLMGYGNKAHGKVTANPGREGRGSVDLEICLRPQIMSTLEADEEFMNFVSMG* |
|  | **Nucleotide sequence (codon-optimised for *S. cerevisiae*)** |
|  | ATGGTGAATCAAGAGGGGATAAAAGTTATAAGTGAGTGCTTTGTGCGTCCAGAGCACGAAGTTGAAGCGGCAAAGAATCCTCTTAGCCTTGGGCCTGTGGACCTTGCGTTCCTATCTATAGACCCAATTCAGAAAGGTTTACTATTTCCCTTCGTAACTAACGACACTCGTCCAGAGATTTCTAGTATTGTCGAACGTCTGAAGAGATCTTTGTCTTTAGCATTAGTTTATTTCTATCCTCTGGCTGGAAGGTTCGAGACCAATAAATATGAGGATGAGCATGCATGTTGGATTTTCCTGGACTGTACAAAAGGTCCAGGCGCCAGGTTAATACACGCTAGCTACGAGGACCTATCTGTCAGCGACATCTTGAGCTCTACAGACGTACACCCTGCTGTGAGATCGTTCTTTGACCTGGGAGAGAGGGTTGTCAATTATGACGGGCACACTAAGGCATTGTTATCCATTCAGGTCACAGAGTTATTGGACGGTGTATTCATCGGGTTTTCTATGAACCATTCAGTGGTAGACGGCACAAGCTTTATACATTTTGTAAGCAGTCTATCCGAGATATTTCGTAGTGACCCTCAAGGTAACGAGGGTCCAGTCAAGATTTCTAGAGTTCCCTTATACAAAATGCACGCGCCAGAGGGGTGCGGCCCTATTAGTAAATTGCCATACCTAGAACACGACGAGTTCATAGTTAGATACGACCCTGGCCCATTGAGAGAGAGGATTTTCCATTTCAGTCCAGAGTCTATGGCCCGTCTAAAGGCCAAGGCTAACGAAGAATGTGGCTCAGGTACACAGGAGATCAGCTCCTACATGGCCTTGTCCAGTCTAGTATGGAAGTCTATCACAAGAGCCTACAACCCAGCGGGACATGACGAGACGTGCTGCTTTACTGCATTGAATGCTCGTCCTAGGCACAATCCCCCGTTGTCAGACGACTACTTCGGCAACATCTTAGTACGTGCTAAAGGGGTTTCAAAAGTCGACGAGTTGCTATCACAGAGCTTGGGGTGGGGTGCTAGAATCTTGAGTCAAGAGGTAAAGAAGCATACGCACGAGACCATCTGTGAGACCTTGAAGATGTTTGCAGAACACCCTTCCGTAATACGTCGTGGATTAAACCAACCGGGTTTCTACTCAGTTGACAACGGCGGGTCCCATCGTTTCGATATGTACGGGCCGGAGTTTGGTCTTGGAAGAGCTGTTGCTGTTTTGATGGGCTATGGTAATAAGGCTCACGGAAAGGTCACAGCAAATCCCGGTCGTGAGGGGAGAGGTTCTGTTGATCTAGAGATTTGCTTAAGGCCCCAAATTATGTCCACTCTAGAGGCAGATGAAGAATTTATGAACTTCGTCTCTATGGGCTAA |
|  | **Nucleotide sequence (codon-optimised for *Y. lipolytica*)** |
|  | CAATCCGCCCTCACTACAACCGATGGTGAACCAGGAAGGAATCAAGGTCATTTCTGAGTGTTTTGTTCGACCCGAGCATGAGGTCGAAGCTGCTAAGAACCCTTTGTCACTGGGTCCGGTGGACCTGGCATTCCTTTCCATCGATCCCATCCAGAAAGGACTCCTGTTTCCTTTTGTTACTAATGACACCAGACCCGAAATCTCATCGATTGTTGAGCGACTGAAGCGATCCCTCTCGCTCGCGCTTGTGTACTTCTATCCCTTGGCCGGCCGATTCGAGACCAATAAGTACGAGGACGAGCACGCGTGCTGGATCTTTCTGGACTGCACGAAAGGACCCGGTGCCCGATTGATCCATGCTTCCTATGAGGACTTGTCCGTGTCCGACATTCTGTCCAGCACAGACGTGCACCCTGCCGTCCGATCCTTCTTCGATCTCGGAGAGCGAGTGGTGAACTATGACGGTCATACCAAGGCACTGCTGTCAATCCAGGTTACCGAGCTTCTTGATGGCGTCTTTATCGGTTTCTCTATGAACCACTCTGTGGTGGACGGCACCTCTTTCATTCACTTTGTGTCTTCGCTGAGCGAAATCTTTCGATCCGATCCCCAGGGTAACGAGGGTCCCGTTAAGATCTCTCGGGTGCCCCTGTATAAGATGCACGCCCCTGAGGGCTGCGGTCCTATCAGCAAGCTTCCCTACCTGGAGCACGATGAATTTATCGTGCGATACGACCCTGGCCCTCTGCGAGAGCGGATTTTCCACTTCTCTCCCGAGAGCATGGCCCGACTTAAGGCTAAAGCTAACGAAGAGTGCGGTTCTGGCACACAAGAGATTAGCTCGTACATGGCCCTGTCATCCCTGGTGTGGAAGTCAATCACCCGTGCCTACAACCCCGCGGGCCACGACGAGACCTGTTGCTTTACCGCTCTGAATGCCCGACCGCGACATAACCCACCATTGTCCGATGACTATTTCGGCAACATCCTTGTTCGAGCTAAGGGAGTCTCTAAAGTCGATGAGCTTCTGTCCCAGTCGCTGGGCTGGGGTGCTCGAATTCTCTCTCAGGAAGTGAAGAAGCACACGCACGAGACTATCTGTGAGACCCTGAAAATGTTCGCAGAGCACCCCTCTGTGATTCGACGGGGCCTCAATCAGCCCGGTTTCTATAGCGTTGACAACGGTGGCTCTCACCGATTTGACATGTACGGCCCAGAATTTGGCCTGGGACGAGCTGTTGCGGTCCTCATGGGCTACGGTAATAAAGCCCACGGTAAGGTCACCGCTAACCCTGGCAGAGAAGGCCGAGGCTCTGTTGACTTGGAGATCTGTCTTCGACCTCAGATCATGTCCACATTGGAAGCCGATGAGGAGTTCATGAATTTTGTCTCCATGGGCTAACTACTCTGGCGTCGATGAGGGA |
| ***Ah*AmaSy1**  [13] | **Amino acid sequence** |
|  | MSHNKESNPNLHVAFYPWFALGHLTSFLRLANKLAERGLQVSYFIPSKTQPKLSPHNHHPNHLTFIPITVPHVDGLPPGAETTNDVPGSAVPLIMTAMDLTQDIIEAHLAQLKPNFVFYDFTYWIPKLGQKLGFKSIHYFTAFISRYGYLAPYKKEGYLPTAADLLRPPPGYPSPIRMKPYEAKIMAGAGKTAFGLGGFTLAERLAVSFIECDAFGVKTCKEMEGEHCKFFEDVFGKPVLLAGPVVPKLPSSKLDEHFDEWLNGFDESSVIFCALGSECSLEINQFHELLLGLELTGRPFLAALKPPKNYKTIESALPEGFASRTRERGIVHEGWVQQQLILQHRSVGCFITHCGVGSLSEAMISKCQVVMIPQAIDQFINARMMSLEWKIGVEIETREDDGWFTREDVHKAITMVMDGESDVGREVRANHAKWRDFILTQGVEDSYISSFIESLQQLLIV* |
|  | **Nucleotide sequence (codon-optimised for *S. cerevisiae*)** |
|  | ATGAGCCACAATAAAGAGAGTAACCCGAACCTTCACGTCGCCTTTTATCCATGGTTTGCACTTGGGCACCTGACTTCATTCCTAAGGTTGGCAAACAAGCTAGCAGAAAGGGGTCTGCAGGTTTCATATTTTATTCCGTCTAAGACACAGCCTAAGTTATCCCCACATAACCATCATCCCAATCATTTAACCTTTATACCAATTACGGTGCCTCACGTTGATGGTTTGCCGCCTGGTGCCGAAACGACTAACGATGTACCCGGTTCGGCTGTTCCCCTGATAATGACTGCAATGGACTTAACCCAGGATATTATTGAGGCCCACTTGGCGCAGTTGAAGCCCAACTTCGTTTTCTATGATTTCACCTACTGGATTCCGAAATTGGGCCAGAAGTTGGGATTCAAGAGTATTCATTACTTCACGGCCTTTATCAGCAGGTACGGTTATTTAGCACCGTACAAGAAAGAGGGATATTTACCCACTGCTGCTGACTTGTTGAGGCCACCCCCAGGATATCCCAGTCCGATTCGTATGAAGCCATACGAGGCTAAAATAATGGCGGGTGCTGGTAAGACCGCTTTTGGATTAGGTGGTTTCACCCTGGCTGAGAGACTAGCCGTATCATTTATCGAGTGCGACGCTTTTGGCGTAAAGACATGTAAAGAGATGGAAGGAGAGCATTGTAAGTTCTTCGAAGATGTGTTTGGGAAGCCAGTACTTCTGGCCGGGCCTGTTGTTCCAAAACTGCCATCAAGCAAGCTAGACGAACACTTCGATGAGTGGCTAAACGGCTTCGACGAGTCATCTGTGATATTCTGTGCTCTTGGATCAGAGTGTTCTCTTGAGATTAACCAGTTCCATGAGCTTCTTTTGGGTTTGGAGTTGACCGGTAGGCCATTCCTAGCAGCCCTAAAGCCACCAAAGAACTATAAAACCATAGAGAGCGCCCTGCCAGAAGGATTTGCTTCAAGAACCCGTGAGAGAGGGATCGTACACGAAGGTTGGGTCCAACAGCAATTAATACTGCAGCACAGGAGTGTGGGCTGCTTCATAACCCACTGCGGCGTAGGCAGTTTGAGCGAGGCTATGATCTCGAAATGTCAGGTCGTTATGATTCCTCAAGCTATAGACCAGTTCATTAACGCTAGAATGATGTCGTTAGAGTGGAAAATAGGAGTCGAAATAGAAACTCGTGAGGACGACGGTTGGTTTACTAGAGAGGACGTTCACAAAGCCATAACAATGGTTATGGATGGCGAGTCCGACGTTGGCAGGGAAGTTAGAGCAAACCACGCAAAATGGAGAGATTTCATACTGACTCAGGGTGTCGAGGACTCCTACATCAGTTCCTTTATCGAATCCTTACAGCAATTATTGATCGTGTGA |
|  | **Nucleotide sequence (codon-optimised for *Y. lipolytica*)** |
|  | ATGTCCCACAACAAGGAGTCGAATCCTAACCTGCACGTGGCTTTCTATCCCTGGTTTGCCCTTGGACATCTGACTTCCTTTCTCCGACTCGCCAACAAGCTGGCCGAGCGAGGCCTTCAGGTTTCCTACTTCATTCCCTCTAAAACCCAACCCAAGCTCTCGCCTCACAACCACCACCCCAACCACCTGACCTTTATCCCTATCACAGTTCCCCATGTCGATGGACTGCCACCCGGCGCGGAAACCACTAACGATGTCCCCGGATCAGCGGTCCCCCTGATCATGACCGCCATGGACCTGACCCAAGACATTATCGAGGCCCATCTGGCACAACTGAAGCCTAATTTCGTCTTTTATGATTTCACTTACTGGATTCCGAAACTTGGTCAGAAGCTGGGATTCAAATCCATTCATTATTTCACCGCCTTCATTTCACGATACGGTTACCTCGCCCCATACAAGAAAGAGGGCTACCTTCCCACTGCCGCCGACCTGCTCCGACCCCCACCCGGTTACCCCTCGCCCATTCGAATGAAGCCTTATGAGGCTAAGATTATGGCTGGAGCCGGAAAGACTGCTTTTGGTCTGGGCGGCTTCACACTCGCTGAGCGATTGGCTGTGTCTTTTATTGAATGTGATGCTTTCGGCGTGAAGACCTGCAAGGAGATGGAAGGCGAACATTGCAAGTTCTTCGAGGACGTGTTCGGCAAACCCGTTCTGCTGGCCGGCCCTGTTGTGCCTAAGCTTCCATCCTCAAAGCTGGACGAGCATTTCGATGAGTGGTTGAATGGCTTTGACGAGTCTTCTGTCATCTTCTGCGCACTGGGTAGCGAGTGCTCGCTTGAGATTAATCAGTTCCACGAACTGCTCCTGGGCCTCGAACTCACAGGCCGACCGTTCCTGGCTGCCCTTAAACCACCCAAGAACTACAAGACAATCGAGAGCGCCCTTCCAGAGGGATTCGCTTCGCGAACGCGAGAGCGTGGAATCGTCCACGAGGGATGGGTGCAACAACAGCTCATTCTGCAACACCGGTCTGTGGGATGCTTTATTACTCACTGCGGCGTCGGCTCTCTGTCAGAGGCTATGATCTCGAAGTGTCAGGTCGTTATGATCCCACAGGCCATCGACCAGTTTATTAACGCTCGAATGATGTCCCTCGAGTGGAAGATTGGCGTCGAGATTGAAACCCGAGAGGATGACGGATGGTTCACTAGAGAGGACGTTCACAAAGCGATCACCATGGTCATGGATGGAGAAAGCGATGTGGGAAGAGAAGTGCGAGCCAATCACGCCAAATGGCGGGACTTCATTCTCACCCAGGGCGTGGAAGACTCCTACATCTCTTCTTTTATTGAGTCCCTGCAGCAACTCCTCATCGTCTGA |
| ***Cq*AmaSy1**  [13] | **Amino acid sequence** |
|  | MSQNKDNQILNVTFYPWFALGHLTSFLRLANKLAERGHNVSYFLPPKTQSKLASHNHYPTHLTFIPIPVPPVEGLPPGAETTNDVPASLGPLIMTAMDMTRDTIESHLVRLKPDIVFYDFTCWMPELGRKHGFKAMHYITAYIARYAYLAPYKKIPGYHPNADDLLTPPPEFPSQSIRMLPQEAEIIAGALKTPFGLGGLTLAERLGVSFRECDAFGVKTCAEMEGEYCKFFEKIFGKPVLLAGPMVPKRPSSELDNYFDDWLNSFRTSSVIYCALGSECALNLNQFQELVLGLELTGRPFLAALKPPMNYQTIESALPEGLAERIKGRGLIHGGWVQQQLILQHPSVGCFITHCGAGSLSEAMVSECQVVLMPQAIDQFISARMMSLEWKVGVEVEKRKNYGLFTKEAVHKAVSLVMEEDSEVGRDVRANHAKWREFILTEGLEDSYISSFIQSLQQLIGS* |
|  | **Nucleotide sequence (codon-optimised for *S. cerevisiae*)** |
|  | ATGTCACAGAACAAGGACAACCAGATCCTAAACGTTACGTTCTATCCTTGGTTCGCATTGGGACATCTTACATCTTTCTTGAGGTTGGCTAACAAGTTGGCGGAGAGAGGCCACAATGTGAGCTATTTCTTGCCACCCAAAACACAGTCCAAACTTGCATCTCATAACCACTATCCAACTCACCTGACATTTATACCTATTCCAGTCCCACCGGTAGAGGGCCTGCCACCTGGGGCGGAGACCACCAATGACGTGCCCGCCAGTTTAGGCCCGTTGATTATGACGGCCATGGATATGACCCGTGACACAATAGAGTCTCATTTGGTCAGGCTTAAGCCAGATATTGTTTTCTACGATTTCACATGCTGGATGCCAGAGCTAGGCCGTAAGCACGGCTTCAAAGCGATGCATTACATAACCGCATACATTGCTCGTTATGCTTATCTAGCCCCCTATAAGAAGATTCCAGGCTATCATCCAAATGCCGATGATCTTCTTACACCGCCCCCTGAGTTCCCCTCTCAGTCGATTCGTATGCTTCCACAAGAGGCCGAGATTATAGCTGGAGCCCTAAAGACGCCATTCGGACTTGGTGGATTGACGCTGGCGGAGAGATTAGGCGTTTCATTCAGAGAGTGTGACGCTTTCGGAGTGAAGACGTGCGCTGAGATGGAAGGTGAGTACTGCAAATTCTTCGAGAAGATCTTCGGAAAGCCTGTGTTATTGGCAGGCCCAATGGTTCCTAAGAGACCTTCTTCTGAGTTGGACAACTACTTTGACGATTGGTTAAATTCCTTCAGAACCTCGAGCGTCATATATTGCGCATTGGGTTCTGAGTGTGCATTGAACCTGAACCAGTTCCAGGAGTTGGTCTTAGGTCTAGAGTTGACTGGAAGACCATTCTTGGCAGCTCTTAAGCCCCCGATGAATTACCAAACGATTGAGAGCGCCCTGCCTGAAGGTCTGGCAGAGCGTATTAAGGGGCGTGGACTTATACATGGCGGCTGGGTCCAACAACAGTTAATACTACAGCACCCTAGCGTAGGATGCTTCATTACACACTGCGGTGCCGGTTCTTTGTCTGAGGCAATGGTGTCGGAATGCCAAGTTGTACTAATGCCTCAAGCCATCGACCAGTTTATCTCGGCGCGTATGATGTCCTTAGAGTGGAAGGTAGGAGTGGAAGTAGAAAAGAGAAAGAACTACGGTCTATTCACTAAAGAAGCAGTTCACAAGGCTGTATCACTAGTAATGGAAGAAGACAGCGAAGTTGGTAGAGACGTTAGGGCCAATCATGCCAAGTGGCGTGAGTTCATTCTAACAGAGGGGTTAGAGGATTCATACATATCTTCGTTTATCCAATCATTACAACAGCTGATCGGATCTTAG |
|  | **Nucleotide sequence (codon-optimised for *Y. lipolytica*)** |
|  | ATGTCCCAAAATAAAGACAACCAGATTCTGAATGTCACCTTTTATCCCTGGTTCGCTTTGGGCCATCTCACATCCTTTCTTAGACTTGCGAATAAACTGGCCGAAAGAGGTCATAATGTTAGCTACTTCCTTCCCCCTAAAACCCAATCAAAGCTGGCGTCCCATAACCACTACCCCACCCATTTGACTTTCATCCCTATTCCGGTGCCACCCGTTGAGGGCCTGCCGCCTGGTGCCGAAACTACGAATGACGTGCCAGCTTCCCTTGGTCCCCTGATTATGACAGCTATGGATATGACTCGAGACACCATCGAGAGCCACCTCGTCCGGCTGAAACCAGATATTGTGTTTTACGACTTCACCTGCTGGATGCCCGAACTCGGTCGAAAGCATGGATTCAAGGCCATGCACTACATTACGGCATATATTGCCCGGTATGCTTACCTCGCACCCTACAAGAAAATCCCGGGATATCACCCAAATGCAGACGACCTTCTTACCCCACCCCCTGAATTTCCAAGCCAATCTATTAGAATGCTTCCTCAGGAAGCTGAAATTATTGCTGGTGCTCTGAAGACACCCTTCGGCCTGGGTGGTTTGACTCTTGCTGAAAGATTGGGAGTCTCGTTCCGTGAGTGTGACGCCTTTGGAGTTAAGACTTGCGCCGAAATGGAAGGTGAATACTGTAAGTTCTTTGAGAAAATCTTTGGTAAACCCGTTCTCCTCGCAGGTCCCATGGTCCCCAAACGACCTTCCTCGGAACTTGATAACTATTTTGATGACTGGTTGAACTCTTTCCGAACCTCGTCGGTCATCTATTGTGCCCTCGGCAGCGAATGCGCATTGAATCTCAATCAATTTCAGGAACTCGTCCTCGGACTCGAGCTGACTGGACGTCCTTTTCTGGCCGCCCTTAAACCGCCCATGAATTATCAAACGATCGAATCCGCCCTTCCCGAGGGCCTCGCCGAACGAATCAAGGGTAGAGGCCTTATTCACGGCGGATGGGTGCAACAACAACTCATTCTTCAGCACCCCTCAGTGGGCTGCTTTATTACACACTGTGGAGCAGGATCGCTTAGCGAAGCTATGGTGTCCGAATGTCAAGTTGTTCTGATGCCACAAGCTATCGACCAATTCATCTCTGCTCGGATGATGTCTTTGGAATGGAAGGTTGGAGTTGAAGTGGAAAAGCGGAAGAACTATGGCCTTTTCACTAAAGAGGCCGTCCACAAAGCCGTGTCGCTTGTTATGGAAGAAGACTCGGAAGTTGGCCGTGATGTCCGTGCAAATCACGCAAAATGGCGTGAATTTATTCTCACTGAAGGACTTGAGGACTCTTACATTTCTTCATTTATTCAATCACTGCAGCAGCTGATTGGAAGCTAA |
| ***Cc*AmaSy1**  **(This study)** | **Amino acid sequence** |
|  | MSSNQDSSVLHVAFYPWFALGHLTSFLRLANKLAERGHKVSYFLPAKTQPKLAPHNHYPNHLTFIPITVPPVDGLPPGAETTNDVPGPSVPLIMTAMDLTRDTIEAHLTHLKPNCVFYDFTHWMPELGRKLGFKSIHYFTAFISRYGYLAPYKKAGYLPTAADLLGPPVGYPSPIRMKAHEAEIMAAAGKNAFGLGGLTLAERLGVSFLECDAFGVKTCEEMEGSHCKYFEEIFGKPVLLAGPVVPKLPSSKLDEYFDGWLNGFGNATVIFCALGSECSLEVNQFQQLVLGLELTGRPFLAALKSPKKCKTIDEALPEGFAKRTRGRGIIYEGWVQQQLILHHPSVGCFITHCGVGSLSEAMISKCQVVMMPQAIDQFINARMMSLEWKIGVEVETREIDGLFTKEAVYKAVSTVMDEESEVGREVRANHAKWREFISMEGVEDSYISSFIESLRQLLG* |
|  | **Nucleotide sequence (codon-optimised for *S. cerevisiae*)** |
|  | ATGAGTTCTAATCAGGATTCTTCGGTACTACACGTAGCCTTTTATCCCTGGTTCGCGTTAGGCCACTTGACCAGTTTCCTAAGATTAGCTAATAAACTGGCCGAGAGAGGCCACAAAGTTTCCTATTTCTTACCCGCCAAGACACAGCCCAAACTAGCACCACACAATCACTACCCCAACCATTTAACATTCATTCCTATCACAGTTCCCCCAGTAGACGGTCTTCCACCAGGTGCCGAAACGACGAACGACGTCCCAGGACCTAGTGTACCCTTGATTATGACTGCGATGGATCTAACGCGTGATACAATCGAAGCCCACTTGACTCATTTGAAGCCTAACTGCGTTTTCTATGACTTTACGCACTGGATGCCAGAGCTGGGTAGGAAACTGGGCTTTAAGTCAATTCACTACTTCACCGCATTCATATCTCGTTACGGATATTTGGCACCTTACAAGAAAGCGGGTTACCTGCCAACGGCAGCCGACTTGCTTGGCCCACCCGTGGGTTACCCTTCACCTATCAGAATGAAAGCTCACGAGGCAGAGATAATGGCTGCCGCAGGTAAGAATGCGTTCGGTTTGGGCGGACTAACTTTAGCAGAGAGATTGGGTGTCTCATTCTTAGAATGTGACGCGTTCGGTGTCAAAACTTGCGAAGAGATGGAAGGATCTCACTGTAAGTATTTCGAAGAGATTTTCGGTAAGCCCGTTCTGTTAGCAGGCCCCGTTGTTCCGAAACTACCTTCCTCTAAGCTGGACGAGTACTTTGATGGCTGGCTAAACGGATTTGGTAACGCCACCGTTATCTTCTGCGCACTGGGGAGTGAGTGTAGCCTAGAGGTCAACCAATTTCAGCAACTTGTCTTGGGTCTAGAGTTAACAGGTAGGCCGTTTCTAGCAGCCTTAAAATCCCCTAAGAAATGCAAGACTATCGACGAGGCCCTTCCCGAGGGGTTTGCTAAGCGTACAAGAGGTAGGGGAATCATCTATGAGGGTTGGGTACAACAACAACTGATACTTCACCACCCTTCTGTGGGGTGTTTCATTACTCACTGCGGAGTAGGGAGCTTGTCAGAGGCAATGATATCTAAATGCCAGGTTGTCATGATGCCTCAGGCGATAGACCAATTCATAAACGCCAGAATGATGTCCTTAGAATGGAAGATTGGAGTTGAGGTAGAAACACGTGAGATCGATGGTTTGTTCACAAAGGAAGCTGTATATAAAGCCGTTTCCACGGTCATGGACGAGGAATCCGAAGTTGGAAGAGAGGTACGTGCTAACCACGCAAAGTGGCGTGAGTTCATCTCCATGGAAGGTGTAGAGGATTCTTACATCTCTTCATTTATTGAGTCCTTGAGGCAATTACTTGGATGA |
|  | **Nucleotide sequence (codon-optimised for *Y. lipolytica*)** |
|  | ATGTCGTCCAACCAGGACTCGTCTGTTCTCCATGTGGCCTTTTATCCCTGGTTTGCTCTCGGCCACTTGACGTCGTTCCTGCGATTGGCTAATAAACTGGCCGAGCGAGGACACAAGGTGTCGTACTTTCTCCCCGCAAAGACTCAGCCCAAGCTGGCACCCCATAACCACTATCCCAATCATCTGACCTTCATCCCTATCACAGTGCCGCCGGTTGACGGACTGCCACCCGGAGCTGAAACTACGAACGATGTCCCTGGCCCTTCCGTCCCGCTGATCATGACCGCCATGGATCTCACTCGAGACACTATTGAGGCCCATCTTACACATCTCAAACCCAATTGTGTTTTCTATGACTTTACCCACTGGATGCCCGAACTGGGCCGAAAGCTGGGTTTCAAGTCCATTCACTACTTCACTGCTTTCATCTCCCGATACGGATATCTCGCGCCGTACAAGAAGGCTGGTTATCTGCCCACCGCTGCAGACCTGCTGGGACCGCCCGTGGGTTACCCTTCCCCTATCAGAATGAAGGCCCATGAGGCCGAAATTATGGCCGCTGCCGGAAAGAATGCATTTGGCCTCGGCGGTCTCACTCTGGCCGAACGACTGGGAGTCTCCTTTCTGGAATGCGACGCCTTCGGCGTGAAGACCTGTGAGGAGATGGAAGGTTCTCACTGTAAATACTTCGAAGAGATCTTTGGAAAGCCTGTTCTGCTGGCGGGTCCCGTGGTCCCTAAACTCCCCTCATCGAAGCTCGACGAATACTTCGATGGTTGGCTGAATGGATTTGGTAACGCGACGGTGATCTTTTGTGCTCTGGGCTCGGAGTGCAGCCTGGAAGTCAACCAGTTTCAACAGCTCGTGCTGGGCCTCGAACTCACGGGCCGTCCCTTTCTGGCCGCCCTCAAGTCCCCTAAGAAATGTAAGACGATTGATGAGGCGCTCCCTGAGGGATTCGCTAAACGAACCCGGGGCCGAGGAATTATTTATGAGGGCTGGGTTCAACAACAACTGATTCTCCACCACCCCTCTGTGGGCTGCTTCATCACGCACTGCGGCGTTGGATCTCTGTCTGAGGCTATGATCTCCAAATGTCAGGTTGTGATGATGCCTCAGGCGATCGACCAATTTATCAACGCCCGGATGATGTCTCTGGAGTGGAAGATTGGTGTCGAGGTGGAAACACGAGAGATCGATGGTCTGTTCACCAAAGAAGCTGTGTACAAGGCCGTTTCGACTGTCATGGACGAAGAGTCAGAAGTGGGCCGAGAGGTCCGGGCCAACCACGCCAAATGGCGAGAATTCATCTCCATGGAAGGTGTCGAGGATTCGTATATTTCGTCGTTCATCGAAAGCCTCAGACAGCTGCTTGGCTAG |
| ***At*UGD1**  ([D7KQP1](https://www.uniprot.org/uniprotkb/D7KQP1/entry))  [14] | **Amino acid sequence** |
|  | MVKICCIGAGYVGGPTMAVMALKCPEIEVVVVDISEPRINAWNSDRLPIYEPGLEDVVKQCRGKNLFFSTDVEKHVFESDIVFVSVNTPTKTQGLGAGKAADLTYWESAARMIADVSKSSKIVVEKSTVPVRTAEAIEKILTHNSKGIEFQILSNPEFLAEGTAIKDLYNPDRVLIGGRDTAAGQKAIKALRDVYAHWVPVEQIICTNLWSAELSKLAANAFLAQRISSVNAMSALCEATGADVTQVAHAVGTDTRIGPKFLNASVGFGGSCFQKDILNLIYICECNGLPEAANYWKQVVKVNDYQKIRFANRVVSSMFNTVSGKKIAILGFAFKKDTGDTRETPAIDVCNRLVADKAKLSIYDPQVLEEQIRRDLSMARFDWDHPVPLQQIKAEGISEQVNVVSDAYEATKDAHGLCVLTEWDEFKSLDFKKIFDNMQKPAFVFDGRNVVDAVKLREIGFIVYSIGKPLDSWLKDMPAVA* |
|  | **Nucleotide sequence (codon-optimised for *S. cerevisiae*)** |
|  | ATGGTAAAGATTTGCTGTATAGGAGCAGGATACGTTGGTGGACCTACCATGGCGGTAATGGCATTGAAGTGCCCAGAGATCGAAGTTGTAGTCGTAGACATATCTGAACCAAGGATAAACGCTTGGAACTCGGATAGACTACCGATATACGAACCAGGACTTGAGGATGTTGTTAAACAATGTAGAGGGAAGAATCTTTTCTTTTCCACAGACGTCGAAAAGCACGTTTTCGAGTCTGATATAGTCTTTGTATCGGTTAATACACCAACGAAGACACAGGGGCTTGGTGCAGGAAAAGCGGCTGATCTGACTTACTGGGAGTCAGCAGCTAGAATGATTGCCGATGTGTCCAAATCATCGAAGATCGTAGTTGAGAAATCTACCGTCCCAGTACGTACAGCAGAAGCAATCGAAAAGATATTGACCCATAATTCGAAGGGAATAGAGTTCCAGATTTTGTCTAACCCGGAGTTCTTAGCCGAGGGAACTGCCATAAAGGACCTATACAACCCTGACCGTGTATTGATAGGTGGGAGAGACACCGCTGCTGGACAGAAAGCAATCAAGGCCCTGAGGGACGTTTATGCACACTGGGTGCCAGTTGAGCAAATAATATGCACCAATTTGTGGTCAGCTGAACTGTCAAAGTTAGCCGCGAATGCATTCTTGGCGCAGAGAATCTCATCTGTCAACGCAATGTCCGCGTTGTGCGAGGCGACAGGAGCCGATGTGACCCAAGTTGCACATGCTGTCGGTACAGATACTCGTATTGGACCCAAGTTCTTGAATGCATCGGTAGGTTTCGGCGGTAGTTGCTTCCAGAAAGATATCTTGAATTTGATTTATATATGTGAGTGCAACGGATTACCTGAAGCGGCTAATTACTGGAAGCAGGTGGTTAAGGTTAACGACTACCAGAAAATACGTTTTGCAAACCGTGTCGTGTCTAGTATGTTTAACACCGTTAGTGGAAAGAAGATCGCAATCCTTGGCTTTGCCTTTAAGAAAGACACTGGGGACACCAGGGAAACACCAGCCATTGACGTCTGCAATAGACTAGTAGCCGACAAGGCAAAACTATCTATCTACGATCCCCAGGTTTTGGAAGAACAGATCAGGAGAGACCTTAGTATGGCAAGGTTCGACTGGGACCATCCAGTTCCATTGCAGCAAATTAAAGCCGAGGGAATCAGTGAACAGGTTAATGTAGTGAGTGACGCTTATGAGGCTACCAAGGACGCACATGGTTTATGCGTCTTGACAGAGTGGGACGAATTCAAGAGTTTGGATTTCAAGAAGATCTTCGACAACATGCAGAAGCCTGCATTTGTTTTCGACGGACGTAATGTGGTGGACGCAGTTAAGTTAAGAGAGATTGGTTTCATTGTCTATTCTATAGGTAAGCCCTTGGACAGCTGGCTTAAAGACATGCCTGCAGTGGCTTGA |

1. **References**

[1] Polturak G, Heinig U, Grossman N, Battat M, Leshkowitz D, Malitsky S, et al. Transcriptome and Metabolic Profiling Provides Insights into Betalain Biosynthesis and Evolution in Mirabilis jalapa. Mol Plant 2018;11:189–204. https://doi.org/10.1016/j.molp.2017.12.002.

[2] Sutor K, Wybraniec S. Identification and Determination of Betacyanins in Fruit Extracts of Melocactus Species. J Agric Food Chem 2020;68:11459–67. https://doi.org/10.1021/acs.jafc.0c04746.

[3] Lukitasari DM, Indrawati R, Chandra RD, Heriyanto, Shioi Y, Botosudarmo THP. pH-dependent stability of major betalains in the encapsulated beetroot extracts (Beta vulgaris L.). J Food Sci 2024;89:2761–73. https://doi.org/https://doi.org/10.1111/1750-3841.17046.

[4] Wybraniec S, Platzner I, Geresh S, Gottlieb HE, Haimberg M, Mogilnitzki M, et al. Betacyanins from vine cactus Hylocereus polyrhizus. Phytochemistry 2001;58:1209–12. https://doi.org/https://doi.org/10.1016/S0031-9422(01)00336-3.

[5] Wybraniec S, Nowak-Wydra B. Mammillarinin: A new malonylated betacyanin from fruits of Mammillaria. J Agric Food Chem 2007;55:8138–43. https://doi.org/10.1021/jf071095s.

[6] Heuer S, Richter S, Metzger JW, Wray V, Nimtzt M, Strack D. Betacyanins from bracts of Bougainvillea glabra. Phytochemistry 1994;37:761–7. https://doi.org/https://doi.org/10.1016/S0031-9422(00)90354-6.

[7] Lystvan K, Kumorkiewicz A, Szneler E, Wybraniec S. Study on Betalains in Celosia cristata Linn. Callus Culture and Identification of New Malonylated Amaranthins. J Agric Food Chem 2018;66:3870–9. https://doi.org/10.1021/acs.jafc.8b01014.

[8] Glitz C, Dyekjær JD, Vaitkus D, Babaei M, Welner DH, Borodina I. Screening of Plant UDP-Glycosyltransferases for Betanin Production in Yeast. Appl Biochem Biotechnol 2025. https://doi.org/10.1007/s12010-024-05100-4.

[9] Glitz C, Dyekjær JD, Mattitsch S, Babaei M, Borodina I. BAHD acyltransferase from dragon fruit enables production of phyllocactin in engineered yeast. FEMS Yeast Res 2025:foae041. https://doi.org/10.1093/femsyr/foae041.

[10] Thomsen PT, Meramo S, Ninivaggi L, Pasutto E, Babaei M, Avila-Neto PM, et al. Beet red food colourant can be produced more sustainably with engineered Yarrowia lipolytica. Nat Microbiol 2023;8:2290–303. https://doi.org/10.1038/s41564-023-01517-5.

[11] Jessop-Fabre MM, Jakočiūnas T, Stovicek V, Dai Z, Jensen MK, Keasling JD, et al. EasyClone-MarkerFree: A vector toolkit for marker-less integration of genes into Saccharomyces cerevisiae via CRISPR-Cas9. Biotechnol J 2016;11:1110–7. https://doi.org/10.1002/biot.201600147.

[12] Holkenbrink C, Dam MI, Kildegaard KR, Beder J, Dahlin J, Doménech Belda D, et al. EasyCloneYALI: CRISPR/Cas9-Based Synthetic Toolbox for Engineering of the Yeast Yarrowia lipolytica. Biotechnol J 2018;13. https://doi.org/10.1002/biot.201700543.

[13] Imamura T, Isozumi N, Higashimura Y, Miyazato A, Mizukoshi H, Ohki S, et al. Isolation of amaranthin synthetase from Chenopodium quinoa and construction of an amaranthin production system using suspension-cultured tobacco BY-2 cells. Plant Biotechnol J 2019;17:969–81. https://doi.org/10.1111/pbi.13032.

[14] Oka T, Jigami Y. Reconstruction of de novo pathway for synthesis of UDP-glucuronic acid and UDP-xylose from intrinsic UDP-glucose in Saccharomyces cerevisiae. FEBS Journal 2006;273:2645–57. https://doi.org/10.1111/j.1742-4658.2006.05281.x.
